# From Phytochemical Complexity to Biological Response: Explainable Modeling of the Antimicrobial Activity of *Cannabis sativa* Resin

**DOI:** 10.3390/molecules31214028

**Published:** 2026-11-09

**Authors:** Dayanna Orosco, Andrea Ramos-Hernández, Mario Romero, María L. Ospina-Castro, Victoria A. Arana, Jolián Andrés Vargas Alzate, Alexander Parody, Carlos Mario Meléndez Gómez

**Affiliations:** 1 Organic and Biomedical Chemistry Research Group, Chemistry Program, Molecular Biology Research Laboratory, Faculty of Basic Sciences, Universidad del Atlántico, Carrera 30 No. 8-49, Puerto Colombia 081007, Colombia; dforozco@mail.uniatlantico.edu.co; 2 Applied Supramolecular Chemistry Research Group, Chemistry Program, Faculty of Basic Sciences, Universidad del Atlántico, Carrera 30 No. 8-49, Puerto Colombia 081007, Colombia; andrearamos@mail.uniatlantico.edu.co (A.R.-H.); marioromero@mail.uniatlantico.edu.co (M.R.); mariaospina@mail.uniatlantico.edu.co (M.L.O.-C.); 3 Science, Education, and Technology Research Group (CETIC), Faculty of Basic Sciences, Universidad del Atlántico, Carrera 30 No. 8-49, Puerto Colombia 081007, Colombia; victoriaarana@mail.uniatlantico.edu.co; 4 CUBIKAN GROUP SAS. Veracruz Farm, La Diamantina District—Km 10, La Argelia Road, Ansermanuevo 762018, Colombia; andres.vargas@cubikangroup.com; 5 Faculty of Engineering, Universidad Libre, Barranquilla 081007, Colombia; alexandere.parodym@unilibre.edu.co

**Keywords:** *Cannabis sativa*, antimicrobial activity, disrupt pre-formed biofilms, phytochemical fingerprint, multivariate analysis

## Abstract

Full-spectrum *Cannabis sativa* resins are complex phytochemical matrices whose antimicrobial activity may vary according to the microorganism, treatment concentration, and biological phenotype evaluated. This study investigated the antibacterial and antifungal activities of *Cannabis sativa* resins and their ability to disrupt pre-formed biofilms of five chemically characterized cannabis resins and evaluated the relative contribution of selected experimental variables to the observed biological responses using inferential statistics and interpretable machine learning. Cannabinoid and terpene profiles were characterized by HPLC-PDA and gas chromatography, respectively. Antimicrobial activity was evaluated against *Staphylococcus aureus*, *Escherichia coli*, *Pseudomonas aeruginosa*, *Acinetobacter baumannii*, *Candida albicans* and *Saccharomyces cerevisiae*. The resins produced microorganism-, concentration-, and phenotype-dependent responses, with complete planktonic growth inhibition observed only for *S. aureus* and more heterogeneous responses against pre-formed biofilms. Generalized linear models identified significant effects of the experimental factors, whereas supervised machine-learning models achieved high predictive performance, particularly for planktonic growth (R^2^ ≈ 0.99). Permutation Importance and SHAP analyses revealed that microbial identity provided the greatest predictive information, followed by treatment concentration, whereas resin identity exerted a smaller, phenotype-dependent contribution. These findings indicate that, within the experimental domain evaluated, microorganism identity and treatment concentration provided greater predictive information than resin identity. Integrating phytochemical characterization with microbiological phenotyping and interpretable modeling of experimental variables provides a useful framework for investigating the biological behavior of complex natural-product matrices.

## 1. Introduction

*Cannabis sativa* L. is one of the oldest cultivated medicinal plants used by humankind; archeological and historical evidence indicates that its use dates back several thousand years, particularly in regions of East Asia, where it was used for therapeutic, ritual, and medicinal purposes [1,2]. Ancient Chinese pharmacopeias already described cannabis-based preparations for the treatment of pain, inflammation, and various neurological disorders. However, throughout the 20th century, the scientific study of cannabis was significantly limited due to legal and regulatory restrictions imposed in many countries [3,4]. In recent decades, changes in legislation and the growing recognition of its pharmacological potential have fostered renewed interest in the systematic research of compounds derived from this plant, including the development of novel analytical strategies for their characterization and quantification [4,5,6].

Several preparations made from flowers, leaves, and resins of *Cannabis sativa* have been used to treat wounds, ulcers, skin infections, abscesses, suppurative lesions, and other conditions characterized by persistent inflammation and signs consistent with infection [2,7]. These applications predate the discovery of microorganisms and the development of the modern concept of antibiotics, they reflect empirical knowledge that attributed to Cannabis the ability to prevent or control the progression of infectious processes and promote tissue healing [3]. Nowadays, numerous pharmacological studies have shown that the cannabinoids, terpenes, and other secondary metabolites present in *Cannabis sativa* exhibit activity against Gram-positive and Gram-negative bacteria, fungi, and microbial biofilms, providing a scientific basis that supports many of these traditional applications [8,9,10]. In this context, the study of chemically characterized cannabis resins provides a relevant framework for evaluating their antimicrobial and antibiofilm properties, thereby contributing to the scientific validation of medicinal use widely documented in traditional medicine [9].

The antimicrobial properties of *Cannabis sativa* have been investigated in the context of its chemically diverse composition, which includes cannabinoids, terpenes, flavonoids, phenolic compounds, and other secondary metabolites. *Cannabis sativa* is characterized by a remarkable diversity of secondary metabolites; more than 500 different compounds have been identified in the plant, including cannabinoids, terpenes, flavonoids, and various phenolic compounds [4,11]. Cannabinoids, particularly Δ^9^-tetrahidrocannabinol (THC) and cannabidiol (CBD), have been extensively studied due to their well-documented pharmacological properties [12,13]. Terpenes, monoterpenes, and sesquiterpenes constitute another important group of bioactive metabolites present in cannabis resins and essential oils; chemical compounds such as myrcene, limonene, α-pinene, linalool, β-caryophyllene, and humulene, among others, are involved in a wide variety of biological activities, these compounds exhibit antimicrobial, antifungal, anti-inflammatory, antioxidant, analgesic, and anticancer properties, suggesting that they play an important role in the therapeutic potential of cannabis extracts [4,9]. For example, the sesquiterpene β-caryophyllene has been described as a selective agonist of the CB2 cannabinoid receptor with anti-inflammatory and antimicrobial activity, while monoterpenes such as α-pinene and limonene have demonstrated antibacterial, bronchodilator, and immune-modulating properties [14].

In addition to their individual effects, it has been proposed that terpenes and other secondary metabolites may functionally interact with cannabinoids to produce combined pharmacological effects; this phenomenon, known as the entourage effect, suggests that the natural mixtures present in full-spectrum cannabis extracts may produce more complex or more potent biological responses than isolated compounds [9]. In this context, it has been proposed that the interaction between cannabinoids, terpenes, and other phytometabolites can modulate various physiological and microbiological processes, including cell membrane permeability [15], inflammatory signaling, modulation of the endocannabinoid system, and antimicrobial activity against pathogenic bacteria and fungi [12]. Previous studies suggest that differences in phytochemical composition may influence the biological properties of cannabis extracts, supporting the importance of chemical characterization when evaluating complex cannabis-derived matrices [4,9].

Despite the growing number of studies demonstrating the antimicrobial potential of cannabinoids and terpenes, most research continues to treat these metabolites as independent entities or to limit itself to the analysis of a few major compounds [16]. Whereas comparatively less information is available on the biological responses of chemically characterized full-spectrum preparations. Studies of isolated phytochemicals are essential for establishing compound-specific biological activity and mechanisms of action, while the evaluation of complex matrices addresses a complementary level of chemical organization in which multiple constituents are simultaneously present. However, studies using complex mixtures do not allow the contribution of individual constituents or their interactions to be established, but instead provide complementary information on the overall biological response of the phytochemical matrix.

The study of complex extracts derived from *Cannabis sativa*, represents a promising line of research. Therefore, this study aimed to evaluate the antibacterial and antifungal activities and the effects on pre-formed biofilms of five chemically characterized *Cannabis sativa* resins. In addition, inferential statistical analysis and interpretable machine-learning approaches were used to examine the relative contribution of microorganism identity, treatment concentration, and resin identity to the observed biological responses.

## 2. Results and Discussion

### 2.1. Chemical Composition of Resins

The chemical composition of *Cannabis sativa* resins is complex and highly variable, resulting from the coexistence of multiple classes of secondary metabolites—primarily cannabinoids and terpenes/terpenoids—whose relative abundance depends on both genotype and cultivation and processing conditions. This chemical diversity not only defines the physicochemical characteristics of the resin but also constitutes a key element for the comparative interpretation of its biological activity without assuming that they represent equivalent phytochemical matrices. In this context, the chemical characterization of the GEN 2, GEN 3, GEN 5, GEN 6, and GEN 7 resins is presented as a descriptive framework to subsequently contextualize the biological assays, without assuming direct causal relationships between individual compounds and antimicrobial activity.

Table 1 summarizes the chemical composition of the five resins analyzed. In the cannabinoid fraction (expressed in g/100 g wet weight), the resins showed a consistently dominant profile of CBD, with values ranging from 27.99 to 80.97 g/100 g, and low levels of Δ^9^-THC (2.16–3.19 g/100 g) (Table 1). In contrast, the cannabinoid acid CBDA showed a wide range (1.61–51.06 g/100 g), suggesting differences among resins in the relative balance between acidic and neutral forms. Minor cannabinoids such as CBDV, CBG, and CBC remained at low levels within the evaluated set.

In the terpene profile, a consistent pattern was observed, characterized by the predominance of sesquiterpenes at high concentrations, notably guaiol (2038.28–4821.02 mg/kg) and β-caryophyllene (2097.38–4178.75 mg/kg) as the major components in all samples, accompanied by α-bisabolol (1360.90–2812.05 mg/kg) and α-humulene (758.27–1472.68 mg/kg). Additionally, variations were observed among resins in monoterpenes and oxygenated terpenoids, including β-myrcene (394.79–978.27 mg/kg), linalool (161.05–630.42 mg/kg), ocimene (112.34–248.44 mg/kg), and limonene (112.79–181.28 mg/kg), as well as caryophyllene oxide (345.55–727.37 mg/kg) and cineol (eucalyptol) (26.13–50.63 mg/kg). These data describe the chemical profile of the resins and their variation among samples, providing context for subsequent biological analyses. In general, the resins evaluated could be differentiated based on their predominant chemical profiles. GEN 2 exhibited a profile characterized by high levels of CBD and sesquiterpenes such as β-caryophyllene and α-bisabolol, while GEN 5 and GEN 7 showed higher relative proportions of CBDA and monoterpenes such as β-myrcene. On the other hand, GEN 6 was notable for its high concentrations of guaiol and linalool, while GEN 3 showed a more balanced composition of neutral and acidic cannabinoids, accompanied by a moderate terpene profile. Overall, these results show quantitative differences in the cannabinoid and terpene profiles of the evaluated resins, providing a chemical framework for contextualizing the biological responses described in the subsequent sections.

The high abundance of terpenes observed in the analyzed resins is particularly significant given the well-documented biological activity of various monoterpenes and sesquiterpenes present in *Cannabis sativa*. Compounds such as β-caryophyllene, α-pinene, β-myrcene, limonene, and linalool have previously been associated with antibacterial, antifungal [17,18], and antibiofilm activity against various pathogenic microorganisms. Previous studies have suggested that these metabolites may disrupt microbial cell membrane integrity due to their lipophilic nature, increasing cell envelope permeability and promoting structural destabilization [19,20]. In addition, specific compounds such as α-pinene and β-myrcene possess notable antibiofilm activity, successfully inhibiting cell aggregation and attenuating the expression of virulence genes in pathogens at sublethal concentrations [21].

Therefore, the evaluated resins shared a broadly similar qualitative phytochemical composition while exhibiting quantitative differences in the relative abundance of individual cannabinoids and terpenes.

### 2.2. Antimicrobial Activity of Cannabis Resins

#### 2.2.1. Gram Negative and Positive Bacteria

The antimicrobial activity of the cannabis resins varied according to bacterial species and resin samples, with marked differences observed between Gram-negative and Gram-positive bacteria. Complete growth inhibition was achieved only in *S. aureus*, while partial inhibition was observed in the Gram-negative bacteria evaluated within the range of experimentally soluble concentrations. Accordingly, activity against Gram-negative bacteria was described using concentration-dependent inhibition percentages, whereas for *S. aureus* it was possible to determine the minimum inhibitory concentration (MIC) (Table 2). Statistical analyses were performed against the growth control using Dunnett’s test, and DMSO controls confirmed that the observed effects were not attributable to the solvent (Supplementary Materials, Table S1).

The integrated analysis of the inhibition profiles using a heatmap (Figure 1) allowed us to identify differential patterns of persistence and magnitude of antibacterial activity among the resins. In general, the responses against Gram-negative bacteria were characterized by a progressive decrease in inhibition as the concentration decreased, although some resins maintained measurable inhibitory activity across a wider range of concentrations. This behavior indicates that the antimicrobial response did not depend only on the maximum inhibition achieved at high concentrations, but also on the relative stability of the inhibitory activity across the dilution gradient.

In general, Gram-negative bacteria exhibited concentration-dependent susceptibility patterns, although the magnitude and persistence of inhibition varied among resins and bacterial species. In *E. coli*, all resins showed moderate inhibitory activity at high concentrations, with GEN5 and GEN7 maintaining inhibition rates above 50% between 256 and 64 µg/mL. In contrast, GEN6 exhibited lower activity against this bacterium, especially at intermediate and low concentrations, while GEN2 exhibited an intermediate profile characterized by partial inhibition mainly restricted to high concentrations. This pattern suggests that activity against *E. coli* was related not only to the initial magnitude of inhibition but also to the persistence of inhibitory activity as the concentration decreased.

The response to *P. aeruginosa* showed a more marked difference among the resins. Although all samples exhibited a progressive decrease in activity at lower concentrations, GEN6 maintained a more stable and sustained inhibitory profile against this bacterium, exhibiting inhibition values greater than 50% down to 32 µg/mL, which differentiated it from the rest of the samples evaluated. On the other hand, GEN2 and GEN3 maintained moderate inhibitory responses in the high concentration range, while GEN5 and GEN7 showed a more pronounced decrease in activity as the concentration decreased. These results indicate differences among the resins in their inhibitory responses against *P. aeruginosa.* Considering the high intrinsic resistance of *P. aeruginosa*, associated with its outer membrane, efflux systems, and low permeability to hydrophobic compounds [22], the partial persistence observed in some resins is particularly relevant from a microbiological perspective. This observation may reflect differences in the integrated phytochemical profiles of the resins, although the contribution of individual cannabinoids or terpenes to the observed activity cannot be established from the present data [18,23].

In *A. baumannii*, the differences among the resins were primarily related to the stability of the inhibitory response across the concentration gradient. GEN3 achieved the highest inhibition at 256 µg/mL, while GEN2 exhibited the most sustained profile, maintaining inhibition percentages above 50% between 256 and 32 µg/mL. In contrast, the other resins showed a progressive decrease in activity as the concentration decreased. This behavior suggests that differences among the resin samples may contribute to the persistence of antibacterial activity across a wide range of concentrations, although the specific contribution of individual phytochemical components cannot be established from the present data.

Although Gram-negative bacteria showed varying levels of susceptibility, *S. aureus* exhibited considerably higher susceptibility to the evaluated resins. Most of the evaluated resins achieved complete growth inhibition over a wide range of concentrations, allowing MIC values to be determined. GEN3 had the lowest MIC (1 µg/mL), followed by GEN5 and GEN7 (2 µg/mL), while GEN2 and GEN6 exhibited values of 8 µg/mL. Additionally, some resins maintained high inhibition percentages even below their respective MICs, indicating measurable antibacterial activity at subinhibitory concentrations. This pattern contrasts sharply with the partial inhibition observed in Gram-negative bacteria is consistent with differences in bacterial susceptibility that may be related to structural differences in the bacterial cell envelope.

The difference observed between Gram-negative and Gram-positive bacteria is consistent with previous reports [17] describing greater susceptibility of Gram-positive microorganisms to cannabinoids and terpene-rich extracts derived from *Cannabis sativa*. This differential response has been attributed primarily to structural differences in the bacterial envelope, particularly the presence of an outer membrane rich in lipopolysaccharides in Gram-negative bacteria, which limits the penetration of hydrophobic compounds [22,24,25]. In contrast, Gram-positive bacteria have been reported to exhibit greater susceptibility to lipophilic metabolites such as cannabinoids and terpenes, potentially facilitating membrane destabilization and antimicrobial activity [25,26].

#### 2.2.2. Yeast

Metabolites derived from *Cannabis sativa*, particularly cannabinoids and terpenes, have shown antifungal activity against various yeasts and opportunistic fungi [27,28]. However, reported susceptibility often varies considerably among fungal species, suggesting that the biological response depends not only on the presence of bioactive compounds but also on the physiological and structural characteristics specific to each microorganism [21,29]. In this context, the resins evaluated exhibited clearly differentiated antifungal patterns against *Candida albicans* and *Saccharomyces cerevisiae*, showing a marked species-dependent response.

Figure 2 and the information contained in Table S2 allowed us to identify significant differences in both the magnitude and persistence of inhibitory activity between the two yeast species. While *C. albicans* exhibited a uniform and limited response across the entire concentration gradient—a behavior consistent with previous reports showing this species’ high tolerance to *Cannabis* extracts, requiring exceptionally high doses to affect its planktonic viability—*S. cerevisiae* exhibited considerably more intense inhibition profiles that depended on the composition of each resin [27,28]. These results indicate that the antifungal activity of the resins was not uniform across yeast species and that susceptibility depended significantly on the species evaluated [27].

In *C. albicans* (Figure 2A), all resins produced relatively low levels of inhibition across the entire concentration range evaluated, without reaching inhibition levels comparable to those previously observed in Gram-positive bacteria [27]. Although most treatments showed statistically significant differences compared to the control, the inhibition percentages remained within moderate ranges and showed relatively narrow variations among resins. This behavior suggests that the resins primarily produced partial growth inhibition against *C. albicans*, rather than strong inhibitory activity. The persistence of moderate responses even at low concentrations indicates that the resin matrices retained measurable inhibitory activity against this yeast; however, the absence of high levels of inhibition suggests lower susceptibility of *C. albicans* under the conditions evaluated [29].

In contrast, *S. cerevisiae* (Figure 2B) exhibited considerably greater susceptibility to the resins, particularly at the highest concentrations tested. The resins achieved inhibition rates exceeding 50%, demonstrating a significantly more intense antifungal response than that observed in *C. albicans*. In addition to the initial magnitude of inhibition, significant differences in the persistence of antifungal activity were identified among the resins. In particular, GEN5 retained significant inhibitory activity across a broader range of concentrations, maintaining high inhibition levels even after successive dilutions. This behavior indicates greater persistence of inhibitory activity against *S. cerevisiae* across the concentration gradient, beyond the maximum inhibition values observed at high concentrations [26].

The marked difference in susceptibility observed between the two yeasts suggests that the antifungal response to the resins may be influenced by physiological characteristics specific to each species [17,29]. Although *C. albicans* and *S. cerevisiae* share a basic eukaryotic organization, they exhibit significant differences in the cell wall composition, membrane regulation, and stress response mechanisms—factors that could influence their interaction with lipophilic metabolites present in the resins [30]. The limited susceptibility observed in *C. albicans* could be related to a greater adaptive capacity against antifungal compounds, a phenomenon previously described in opportunistic yeasts with high phyisiological plasticity [21,28].

When these results are considered alongside the chemical characterization of the resins (Table 1), GEN5—the resin with the most persistent antifungal profile against *S. cerevisiae*—also exhibited high concentrations of guaiol and linalool, as well as notable levels of β-caryophyllene and caryophyllene oxide. Various studies have linked these terpenes to alterations in membrane integrity, changes in cell permeability [31], and disruptions to metabolic processes in fungal microorganism [32]. However, given the phytochemical complexity of *Cannabis sativa*, the observed associations should be interpreted descriptively rather than as direct causal relationships, as antifungal activity likely depends on multifactorial interactions among multiple secondary metabolites [33].

### 2.3. Activity of Cannabis Resins Against Pre-Formed Biofilms

Biofilm formation is a microbial phenotype closely associated with resistance to and tolerance of antimicrobial agents, as well as with the persistence of chronic and recurrent infections [34,35]. These structures consist of organized cellular communities immersed in an extracellular matrix composed primarily of polysaccharides, proteins, and extracellular DNA, which limits the diffusion of antimicrobial compounds, promotes cell survival, and creates distinct physiological microenvironments [36,37,38]. As a result, microorganisms associated with biofilms may exhibit reduced susceptibility to conventional treatments compared to their planktonic counterparts. This phenomenon has been extensively documented in Gram-negative and Gram-positive bacteria, as well as in clinically significant yeasts, where biofilm formation represents a determining factor in microbial colonization [34,35,39], persistence, and virulence. Various studies have shown that cannabinoids and terpenes can interfere with key stages of biofilm development, including cell adhesion, extracellular matrix production, membrane integrity, and intercellular communication systems [28,40]. Considering the diversity of *Cannabis sativa* resins and the physiological differences among the microorganisms evaluated, their activity against pre-formed biofilms was assessed across the tested microbial species.

#### 2.3.1. Gram Negative and Positive Bacteria

To minimize the contribution of growth inhibition to the reduction in pre-formed biofilm biomass, the concentrations evaluated in the pre-formed biofilm assays were selected based on the growth inhibition results, prioritizing concentrations that did not produce a marked inhibitory effect. For *S. aureus*, where complete growth inhibition was observed and an MIC was determined within the tested range, concentrations based on the MIC were also included.

The activity of cannabis resins against pre-formed biofilms depended on both the genotype evaluated and the bacterial species, showing a dose-dependent response with clearly differentiated patterns among microorganisms (Figure 3). Although all resins demonstrated the ability to reduce pre-formed biofilm biomass, the magnitude of the response and sensitivity to the evaluated concentrations varied considerably. These results suggest that the observed activity cannot be attributed exclusively to a general antimicrobial effect but likely involves specific interactions with biological processes related to cell adhesion, extracellular matrix production, and the regulation of biofilm development [37,41,42,43].

Among Gram-negative bacteria, marked differences in response to the resins were observed. In *E. coli*, GEN7 exhibited the earliest response and the highest levels of inhibition across the range of concentrations evaluated, suggesting greater antibiofilm activity across the concentration range evaluated [34]. However, this pattern was not observed in *P. aeruginosa*, where GEN6 exhibited the highest activity, while GEN5 showed the lowest response. The inversion in the hierarchy of activity between the two species indicates that susceptibility to the resins does not depend exclusively on structural characteristics shared by Gram-negative bacteria, such as the presence of an outer membrane, but also on differences in the regulatory systems associated with biofilm formation and maintenance [34].

Differential behavior was observed in *A. baumannii*, where GEN3 consistently showed the highest levels of inhibition, while GEN6 exhibited one of the limited responses. This result is particularly relevant because GEN6 was the most active resin against *P. aeruginosa*, demonstrating that the antibiofilm activity of resins is highly dependent on the biology of each microorganism [40]. The differences observed among the three Gram-negative species could be related to variations in the composition of the extracellular matrix, surface adhesion mechanisms, biofilm architecture, and the cellular signaling systems involved in their development [34,38,44].

On the other hand, *S. aureus* exhibited the most striking susceptibility pattern. In contrast to Gram-negative bacteria, in which concentrations in the range of several µg/mL were required to achieve moderate levels of inhibition, this bacterium responded to considerably lower concentrations. The high susceptibility observed suggests that the pre-formed *S. aureus* biofilms were particularly responsive to the resin matrices under the experimental conditions evaluated. Among the tested resins, GEN3 produced the greatest reduction in pre-formed biofilm biomass, followed by GEN7. This finding is consistent with previous studies reporting that phytocannabinoids such as CBD and CBG exhibit activity against *S. aureus* and established staphylococcal biofilms [40,43,45]. However, although these compounds were detected in the evaluated resins, the present experimental design does not allow the differences observed among GEN matrices to be attributed to specific cannabinoids or terpenes.

Among Gram-negative bacteria, the greatest reductions in pre-formed biofilm biomass were observed for [44,45] in *P. aeruginosa* with GEN 6 at 8 µg/mL (50.29 ± 5.71), whereas for *E. coli*, the best result was observed with GEN 7 at 16 µg/mL (43.2 ± 9.78) and for *A. baumannii* GEN 3 at 16 µg/mL (42.79 ± 6.55) [45,46]. For *S. aureus*, GEN3 produced the greatest reduction in pre-formed biofilm biomass at 0.25 µg/mL (47.76 ± 10.93%), the fact that the resin associated with the greatest response differed among bacterial species indicates that the activity against pre-formed biofilms was dependent on the specific microorganism–resin combination rather than being uniformly associated with a particular resin matrix [32,39,41].

Previous studies have reported that cannabinoids and terpenes have been associated with multiple mechanisms of action relevant to biofilm control, including alterations in membrane integrity, increased cell permeability, inhibition of quorum sensing systems, reduced extracellular matrix production, and disruption of bacterial adhesion processes [40,45]. These mechanisms provide a biological context for interpreting the observed activity but were not directly investigated in the present study.

#### 2.3.2. Yeast

The ability of yeasts to produce biofilms depends on a complex interaction between cellular adhesion mechanisms, extracellular matrix production, and physiological adaptation to sessile growth [46,47]. These structures constitute an important factor in microbial persistence because they promote tolerance to antimicrobial agents and adverse environmental conditions [48]. In this context, differences in the response of pre-formed yeast biofilms to the evaluated resins should be considered in relation to the distinct biological and structural characteristics of the biofilms formed by each species [47].

The results revealed a concentration-dependent reduction in pre-formed biofilm biomass in both yeasts, although with notable differences in the magnitude of the response and in the relative performance of the resins (Figure 4). In *C. albicans*, GEN 6 at 32 µg/mL exhibited the highest reduction in pre-formed biofilm biomass, achieving a reduction exceeding 70%, while GEN 3 and GEN 7 showed intermediate responses and GEN 5 exhibited the lowest efficacy. In contrast, a different pattern was observed in *S. cerevisiae* where GEN 6 maintained high activity, but GEN 2 showed comparable behavior at the highest concentrations, while GEN 3 quickly reached a plateau near 40% biomass reduction. These differences indicate that the response to the resins does not depend solely on the applied concentration, but also on the specific physiology of each yeast and the way in which their sessile communities respond to complex bioactive metabolites [36,39].

The marked susceptibility of *C. albicans* to some of the resins evaluated may be related to the biological complexity of its biofilms [39]. Unlike other yeasts, this species has the ability to develop filamentous structures and undergo morphological transitions between yeast-like and hyphal forms—processes closely associated with biofilm maturation, extracellular matrix production, and increased tolerance to antimicrobial agents [39,47]. Several studies have shown that terpenoid compounds present in essential oils and plant extracts can alter the integrity of fungal membranes, affect cell adhesion mechanisms, and even modulate processes related to the formation of filamentous structures—mechanisms that could contribute to the behavior observed in this species [49,50]. In contrast, *S. cerevisiae* biofilms exhibit a less complex structural organization and do not rely as heavily on morphological differentiation processes as those described for *C. albicans* [36].

### 2.4. Statistical and Machine-Learning Analysis of Factors Associated with Antimicrobial Activity and Activity Against Pre-Formed Biofilms

#### 2.4.1. Experimental Factors Associated with Growth Inhibition

Multifactorial ANOVA analysis indicated that the response was strongly influenced by the concentration gradient, although this does not act in isolation. The concentration covariate was highly significant (F = 732.69; *p* = 0.0000), and even after controlling for this effect, significant differences remained between microorganisms (F = 205.40; *p* = 0.0000) and between resins (F = 7.84; *p* = 0.0000). Therefore, these results support the conclusion that growth inhibition is not explained solely by concentration but is also influenced by microorganism identity and the resin evaluated. This interpretation is reinforced by the significant microorganism–resin interaction (*p* = 0.0119).

Overall, these findings indicate that growth inhibition was significantly associated with treatment concentration, microorganism identity, and resin identity, with the significant microorganism–resin interaction showing that differences among the evaluated resin matrices varied across microbial species [20].

#### 2.4.2. Experimental Factors Associated with Pre-Formed Biofilm Biomass Reduction

The multifactorial model applied to pre-formed biofilm biomass showed that concentration explained a very large proportion of the variation (very high F-value; *p* < 0.0001) and, even after controlling for that gradient, significant effects of both microorganism and resin persisted (both *p* < 0.0001). Of particular relevance for biological interpretation, the microorganism–resin interaction was also significant (F = 16.61; *p* < 0.0001), indicating that differences among the evaluated resin matrices varied according to the microorganism. Overall, these findings demonstrate a heterogeneous response of pre-formed biofilms associated with treatment concentration, microorganism identity, and resin identity.

Collectively, the findings presented in Section 2.3.1 and Section 2.3.2 support the conclusion that the antimicrobial and pre-formed biofilm responses of *Cannabis sativa* resins cannot be interpreted as simple concentration-dependent responses or attributed to the action of individual cannabinoids or terpenes. Instead, both phenotypes are consistent with an emergent response arising from the interplay between the phytochemical fingerprint of each resin and the physiological characteristics of the target microorganism. While the previous sections discuss the biological responses observed in planktonic growth inhibition and pre-formed biofilm biomass reduction separately, these responses likely represent complementary manifestations of the biological activity of the resin matrices. Therefore, an integrated statistical analysis was performed to simultaneously evaluate the relative contributions of concentration, microorganism identity, resin composition, and their interactions, with the aim of identifying the principal determinants governing the overall antimicrobial and pre-formed biofilm responses of the evaluated cannabis resins.

#### 2.4.3. Integrated Statistical Analysis of Antimicrobial and Pre-Formed Biofilm Responses

The antibacterial, antifungal, and pre-formed biofilm assays generated a multidimensional dataset in which the biological responses varied according to resin identity, microorganism species, treatment concentration, and the biological phenotype evaluated. Since these experimental factors can act concurrently, their individual effects cannot be adequately interpreted using univariate comparisons. Therefore, an integrated statistical framework was applied that combined generalized linear models (GLM) and machine learning approaches to quantify the contribution of each factor to the observed biological responses and identify the variables with the greatest predictive power.

The combined assessment of growth inhibition and pre-formed biofilm biomass reduction allowed for the analysis of two complementary physiological phenotypes of microbial behavior in response to *Cannabis sativa* resins. While growth inhibition reflects the ability of the treatments to suppress the proliferation of planktonic cells, biofilm inhibition assesses their effect on a highly organized multicellular phenotype associated with persistence, antimicrobial tolerance, and virulence. Integrating both responses within a single statistical framework allowed us to evaluate the relative influence of resin identity, microbial species, and treatment concentration on complementary biological processes. Since each resin constitutes a complex phytochemical matrix, the analyses were designed to evaluate the biological responses of the complete resin matrix, without inferring the contribution of individual metabolites, as this cannot be determined with the experimental design used.

This tendency is clearly evident in Figure 5, where all resins shifted the absorbance to lower values compared to the control in both assays, while the confidence intervals showed considerable overlap among treatments. Consequently, the differences observed among the resins were mainly related to the magnitude of the biological response rather than to the presence or absence of inhibitory activity, since none of the resin matrices was completely inactive across the evaluated microbial panel. These results suggest that, when activity is analyzed holistically, the variations among the resins account for only a portion of the observed response, making it necessary to explore the influence of other experimental factors, particularly microorganism-specific susceptibility.

Microorganism-specific susceptibility emerged as one of the main determinants of the biological response to *Cannabis sativa* resins (Table 3). In the growth inhibition assay, the average reduction ranged from 14.1% to 75.6%, indicating marked heterogeneity among the species evaluated. In contrast, the reduction in pre-formed biofilm biomass showed a narrower range of response (18.5–23.8%), indicating that differences among microorganisms were considerably smaller when this phenotype was evaluated. Therefore, these results indicate that microorganism-specific susceptibility contributes differently to the two biological responses, with substantially greater interspecies variability observed for planktonic growth inhibition than for the reduction in pre-formed biofilm biomass.

Figure 6 illustrates these differences more clearly. During planktonic growth, *Staphylococcus aureus* exhibited the highest average inhibition, while *Candida albicans* showed the lowest sensitivity to the treatments, defining the extremes of the response observed among the microorganisms evaluated. Meanwhile, *Acinetobacter baumannii*, *Escherichia coli, Pseudomonas aeruginosa,* and *Saccharomyces cerevisiae* formed a group with intermediate susceptibilities. In the pre-formed biofilm assay, however, the differences between species were less pronounced, and the responses tended to cluster within a relatively narrow range, suggesting that reduction in pre-formed biofilm biomass was more homogeneous when pre-formed biofilms were evaluated than when the growth of planktonic cells was analyzed.

A comparison across both assays reveals that the biological response was not determined exclusively by the treatment applied but was also influenced by the physiological characteristics associated with the microbial phenotype under evaluation. While planktonic growth reflected marked differences in microorganism-specific susceptibility of the species, pre-formed biofilm biomass reduction exhibited a relatively more uniform response to the resins. This behavior suggests that the mechanisms regulating cell proliferation and those involved in the maintenance of pre-formed biofilm biomass respond differently to treatment, highlighting the importance of considering both phenotypes to obtain a more complete characterization of the biological potential of *Cannabis sativa* resins [51].

Treatment concentration was another factor associated with the biological response of *Cannabis sativa* resins, although its effect differed between the two phenotypes evaluated. In the growth-inhibition assay, increasing treatment concentration was associated with a progressive decrease in absorbance and a concomitant increase in inhibitory activity, consistent with a concentration-dependent response. In contrast, the response of pre-formed biofilms showed greater variability across the concentration range, indicating that the reduction in pre-formed biofilm biomass did not follow the same concentration–response pattern observed for planktonic growth inhibition. These differences indicate that planktonic growth inhibition and reduction in pre-formed biofilm biomass represent distinct biological responses to resin treatment [10].

#### 2.4.4. Statistical Significance and Predictive Performance of Experimental Factors

In order to determine whether the differences observed in the descriptive analysis remained statistically significant after simultaneously considering the microorganism, the resin type, and the treatment concentration, a generalized linear model (GLM) was fitted using the absorbance data obtained from the growth inhibition and pre-formed biofilm assay.

The generalized linear model (GLM) revealed that the microorganism and concentration significantly affected absorbance in both assays (*p* < 0.0001). In contrast, resin identity did not significantly influence growth inhibition (F = 0.43; *p* = 0.7889), whereas it remained a significant factor during the pre-formed biofilm assay (F = 21.79; *p* < 0.0001). These results indicate that the contribution of resin type depends on the microbial phenotype being evaluated, whereas the microorganism and concentration consistently explained a significant proportion of the observed variability (Table 4). As a sensitivity analysis addressing the geometric nature of the dilution series, the same GLM models were re-fitted using log2-transformed concentration; in all four models (growth and biofilm, absorbance and % inhibition), model fit improved (R^2^ increased by approximately 0.11–0.18) while concentration remained the strongest and most significant factor (*p* < 0.0001) and the pattern of statistical significance for microorganism and resin identity was unchanged, indicating that the qualitative conclusions of the study do not depend on the scale used for concentration.

However, although the GLM made it possible to identify factors with statistically significant effects, statistical significance itself does not quantify the relative contribution of each experimental factor to the biological response. For this reason, *machine learning* models were used to evaluate the predictive importance of the microorganism, the concentration, and the resin type.

Tree-based machine learning algorithms consistently outperformed linear and kernel-based approaches in both biological assays (Table 5). In the growth inhibition assay, all assembled tree models achieved excellent predictive performance (test R^2^ ≈ 0.99), indicating that the experimental variables explained practically all of the variability in the response. A leave-one-replicate-group-out cross-validation (GroupKFold, k = 6), in which no single replicate was shared between p’ training and test folds, produced virtually identical performance (e.g., R^2^ = 0.988 for growth % inhibition and R^2^ = 0.770 for biofilm % inhibition, versus 0.987 and 0.775 under the original split), confirming that the reported R^2^ values are not inflated by data leakage between replicates. In contrast, the reduction in pre-formed biofilm biomass exhibited lower predictive power (test R^2^ ≈ 0.94–0.95), indicating that the observed pre-formed biofilm response was less fully captured by the experimental variables included in the model. Meanwhile, Ridge regression consistently showed the lowest performance, indicating that the linear relationships captured by Ridge regression were insufficient to describe the observed response patterns. Since several assembled tree models exhibited comparable predictive performance, for each response variable, the best-performing algorithm was selected for subsequent Permutation Importance, SHAP, and Partial Dependence analyses.

#### 2.4.5. Relative Contribution of Experimental Factors

Although the generalized linear model (GLM) allowed us to identify the experimental factors with statistically significant effects on the biological responses, statistical significance does not necessarily reflect the relative importance of those factors for the model’s predictive performance. Therefore, Permutation Importance analysis was performed using the selected tree-based models to assess the relative predictive importance of microorganism identity, resin identity, and treatment concentration in predicting growth inhibition and pre-formed biofilm biomass reduction.

The Permutation Importance analysis revealed a clear hierarchy among the experimental factors in terms of their predictive importance for the absorbance-derived responses (Figure 7). In both biological phenotypes, microorganism identity was the primary predictor, while concentration made a secondary contribution, and resin identity consistently showed the lowest predictive importance. Although the relative importance of concentration differed between growth inhibition and pre-formed biofilm biomass reduction, the general pattern remained unchanged, with microorganism identity contributing most strongly to the predictive performance of the models.

These results complement those obtained using GLM, demonstrating that statistical significance does not necessarily translate into predictive relevance. Although resin identity remained a statistically significant factor in the pre-formed biofilm biomass reduction model, its contribution to prediction was considerably lower than that of the microorganism and concentration. Overall, these findings indicate that, within the fitted models, microorganism identity was the most influential predictor of the biological responses to *Cannabis sativa* resins, followed by treatment concentration, whereas differences among resin matrices made a comparatively smaller contribution to the prediction of the observed responses.

#### 2.4.6. SHAP Interpretation of the Experimental Variables

To get a deeper understanding of the predictive models, we applied the SHAP (*SHapley Additive exPlanations*) analysis, a tool that identifies the contribution of each category within the experimental variables to the predicted response. Unlike *Permutation Importance* analysis, which quantifies the overall importance of each predictor, SHAP allows us to establish which microorganisms, concentration levels, and resin types are primarily responsible for the predictions generated by the selected machine learning models.

To identify the specific categories responsible for the predictions generated by the models, SHAP (*SHapley Additive exPlanations*) analysis was applied using the best-performing machine learning algorithms (Figure 8). The results showed that the influence of microorganisms on the predicted response depended on the phenotype being evaluated. In the growth inhibition assay, *C. albicans* and *S. aureus* were the species that contributed greatly to the prediction of absorbance. In contrast, for pre-formed biofilm biomass reduction, *S. aureus* emerged as the primary determinant of the model, followed by *P. aeruginosa*, *E. coli*, and *A. baumannii*, while *C. albicans* and *S. cerevisiae* showed a considerably lower influence. These results demonstrate that the microorganisms that dominate the biological response differ between the planktonic growth state and the sessile phenotype, reflecting the physiological characteristics associated with each microbial state.

Regarding the identity of the resins, the SHAP analysis corroborated the tendency previously observed using *Permutation Importance*. Although differences were detected among the matrices evaluated—particularly in the pre-formed biofilm response, where GEN 3 showed the highest relative contribution—the magnitude of this contribution was considerably lower than that associated with microorganism. Consequently, identity and concentration differences among the resins made a comparatively smaller contribution to the model predictions than microorganism identity and treatment concentration.

Meanwhile, partial dependence analysis allowed us to visualize the effect of concentration on the model’s predictions. In both assays, an increase in concentration promoted a progressive decrease in absorbance and a concomitant increase in the percentage of inhibition, confirming a dose–response relationship. However, this behavior was more uniform during growth inhibition than in the pre-formed biofilm assay, where the response curve exhibited greater variability. This pattern is consistent with the tendencies observed in Figure 9 and indicates that the concentration–response relationship was more variable for pre-formed biofilm biomass reduction than for planktonic growth inhibition.

## 3. Materials and Methods

### 3.1. General Information

All reagents were acquired from Merck (Darmstadt, Germany) and Sigma-Aldrich (St. Louis, MO, USA). The reference bacterial strains (*Pseudomonas aeruginosa* ATCC 15442, *Acinetobacter baumannii* ATCC 19606, *Escherichia coli* ATCC 25922 and *Staphylococcus aureus* ATCC 25923) and the yeast strains (*Candida albicans* ATCC 10231 and *Saccharomyces cerevisiae* ATCC 9763) were supplied by MICROBIOLOGICS (St. Cloud, MN, USA). Samples of GEN 2, GEN 3, GEN 5, GEN 6, and GEN 7 resins were supplied by CUBIKAN GROUP SAS. Address: Finca Veracruz, Vereda La Diamantina—Km 10, La Argelia Road (Ansermanuevo, Valle del Cauca), Colombia. Contact: Business Office—Phone: 606 8910050—Email: info@cubikangroup.com.

### 3.2. Cannabis Resin Extraction

The five full-spectrum resins were obtained from *Cannabis sativa* L. cultivars officially registered in the Colombian National Registry of Commercial Cultivars (ICA). The experimental resin codes corresponded to the following cultivars: GEN2, ANDICA CBD; GEN3, EMBERA; GEN5, YOCOTO; GEN6, LIMON CBD; and GEN7, VALLE DE LOS UMBRAS. All source cultivars were classified as non-psychoactive, CBD-predominant cannabis. Detailed information on cultivar registration, geographical origin, source-flower cannabinoid profile, and cultivation conditions is provided in Supplementary Table S17. *Cannabis sativa* resins were obtained from dried inflorescences through etanol extraction, followed by solid–liquid separation, winterization, sequential filtration, and solvent removal under reduced pressure. The concentrated resins were homogenized, packaged, and stored under controlled conditions until their chemical characterization and biological evaluation. Residual solvent content was monitored in accordance with the recommendations of the ICH Q3C and USP <467> guidelines.

### 3.3. Analysis of Resins Chemical Composition

The cannabinoid profile of the *Cannabis sativa* resins was quantitatively determined using high-performance liquid chromatography coupled to a photodiode-array detector (HPLC-PDA), following a laboratory-adapted procedure based on AOAC Official Method 2018.10. For sample preparation, 200.7 mg of each resin was extracted to a final volume of 20 mL. Both the undiluted extract and an aliquot subjected to a 20-fold dilution were analyzed to accommodate the different concentration ranges of major and minor cannabinoids. Chromatographic separation was performed under reversed-phase conditions using a Kinetex C18 column (100 × 3.0 mm, 1.7 µm particle size) maintained at 35 °C. The mobile phase consisted of water containing 0.085% phosphoric acid and acetonitrile containing 0.085% phosphoric acid in a 30:70 (*v*/*v*) ratio, delivered at a flow rate of 1.6 mL min^−1^. A 5 µL aliquot was injected, and detection was performed using a PDA detector at 220 nm with a bandwidth of 4 nm. Chromatographic data were acquired and processed using LabSolutions software versión 5.114, with an analytical acquisition window of approximately 10 min.

### 3.4. Strains and Culture Conditions

The bacteria were cultured at 37 °C for 24 to 48 h on solid and liquid media. *P. aeruginosa* was initially maintained on cetrimide agar and then cultivated in LB broth. *E. coli* and *A. baumannii* were activated on MacConkey agar and subsequently transferred to LB broth. *S. aureus* was first cultured on blood agar and then in BHI broth. Bacterial growth was monitored by measuring the optical density at 600 nm using a Multiskan SkyHigh microplate reader (Thermo Scientific, Waltham, MA, USA). For yeasts, YPD and Sabouraud dextrose agar were used. *C. albicans* were incubated at 37 °C for 3 days, and *S. cerevisiae* at 30 °C for 5 days until characteristic colonies formed. Each strain was then transferred to 2% Sabouraud broth. Growth was measured by optical density at 540 nm for *C. albicans* and 600 nm for *S. cerevisiae*, using the Multiskan SkyHigh reader (Thermo Scientific, Waltham, MA, USA).

### 3.5. Antimicrobial Assays

Antimicrobial activity was evaluated using a broth microdilution assay in 96-well microplates, based on previously published methodologies with modifications. The assays were performed using serial twofold dilutions of the cannabis resins, and microbial growth was monitored spectrophotometrically after incubation. The experimental procedures for bacterial and yeast assays are described separately below.

#### 3.5.1. Antibacterial Assays

For the antibacterial assays, 125 µL of an inoculum adjusted to 0.5 McFarland (OD_600_ ≈ 0.1) were added to 96-well microplates, followed by 125 µL of Mueller–Hinton broth containing the cannabis resins at serial twofold concentrations ranging from 256 to 0.25 µg/mL. The microplates were incubated at 37 °C with agitation for 24 h, and absorbance was measured at 600 nm every 2 h using a Multiskan SkyHigh microplate reader (Thermo Scientific). Each concentration was evaluated in duplicate on three independent experimental days (*n* = 3).

Gentamicin was included as the positive antibacterial reference control. Untreated cultures were used as growth controls, while DMSO-containing cultures were included as vehicle controls. The minimum inhibitory concentration (MIC) was defined as the lowest tested concentration at which complete inhibition of microbial growth was observed based on the absorbance measurements under the experimental conditions. When complete growth inhibition was not achieved within the tested concentration range, no MIC value was assigned, and the antimicrobial response was reported as the percentage of growth inhibition relative to the untreated growth control.

The MIC values of the cannabis resins and the reference antimicrobial agents are summarized in Table 2. Minimum bactericidal concentrations (MBCs) were not determined in this study.

#### 3.5.2. Antifungal Assays

For the yeast assays, inoculum equivalent to 0.5 McFarland were prepared and exposed to the cannabis resins at serial twofold concentrations ranging from 32 to 0.0315 µg/mL in 96-well microplates. *Candida albicans* were incubated at 37 °C and monitored at 540 nm, whereas *Saccharomyces cerevisiae* was incubated at 30 °C and monitored at 600 nm. Both assays were conducted for 24 h, and each concentration was evaluated in duplicate over three independent experimental days.

Ketoconazole was included as positive antifungal reference controls. Untreated cultures were used as growth controls, while DMSO-containing cultures were included as vehicle controls. The minimum inhibitory concentration (MIC) was defined as the lowest tested concentration at which complete inhibition of microbial growth was observed based on the absorbance measurements under the experimental conditions. When complete growth inhibition was not achieved within the tested concentration range, no MIC value was assigned and the response was reported as the percentage of growth inhibition relative to the untreated growth control. Minimum fungicidal concentrations (MFC) were not determined in this study.

### 3.6. Activity Against Pre-Formed Biofilms

The activity against pre-formed biofilms assay was performed in 96-well polystyrene microplates, according to [52,53], with some modifications. LB broth was used for *P. aeruginosa*, TSB broth supplemented with 1% glucose was used for *E. coli* and *S. aureus*, and TSB broth with 0.5% glucose was used for *A. baumannii*. A 1:100 inoculum of cultivars to OD_600_ was used. For *C. albicans*, Sabouraud broth supplemented with 2% glucose was used, and for *S. cerevisiae*, YPD broth with 2% glucose was used. The bacteria were incubated under static conditions at 37 °C for 24 h; the yeasts were incubated at 37 °C (*C. albicans*) and at 30 °C (*S. cerevisiae*) for 48 h. After incubation, non-adherent cells were removed, and the cells were washed twice with PBS. The resins were then added at concentrations determined from the growth assays, prioritizing concentrations with no marked inhibitory effect on microbial growth and, only when complete growth inhibition was observed (*S. aureus*), concentrations based on the MIC. In this way, the activity against pre-formed biofilms was performed while minimizing interference from effects associated with growth inhibition. Then, the plates were incubated again under the same conditions. Subsequently, the treatments were removed, the plates were washed twice with PBS, and 200 µL of 0.1% crystal violet was added for 20 min. After discarding the dye, the plates were washed three times with sterile water and allowed to dry. Finally, ethanol (200 µL) was added to solubilize the bound dye, the mixture was shaken for 15 min, and the absorbance was measured at 570 nm. The reduction in pre-formed biofilm biomass was expressed as a percentage relative to the untreated biofilm control.

### 3.7. Statistical Analyses

#### 3.7.1. Descriptive and Inferential Statistical Analysis

Each experiment was conducted independently at least three times, and the results are expressed as the mean ± standard deviation (SD). For the growth inhibition and pre-formed biofilms assays, differences between the control group (untreated) and the treated groups were evaluated using a one-way analysis of variance (One-way ANOVA), followed by Dunnett’s multiple comparison test, using the control group as a reference. These analyses were performed using GraphPad Prism software, version 10.

#### 3.7.2. Generalized Linear Models (GLM)

To simultaneously evaluate the effect of microorganism identity, resin identity (GEN), and treatment concentration on absorbance and the percentage of inhibition, generalized linear models (GLM) were fitted. The models included microorganism and resin as categorical factors and treatment concentration as a quantitative covariate. The significance of the factors was determined using analysis of variance with Type III sums of squares, calculating the F, *p*, R^2^, and adjusted R^2^ values. A significance level of *p* < 0.05 was adopted. As treatment concentration followed a two-fold serial dilution series, all GLM models were additionally re-fitted using log2-transformed concentration as a sensitivity analysis; results of this comparison are reported in the Results section. These analyses were performed using StatGraphics Centurion software, versión 18.

#### 3.7.3. Machine Learning Models

To evaluate the predictive power of the experimental variables on absorbance and the percentage of inhibition, ten supervised machine learning algorithms were implemented: Ridge Regression, K-Nearest Neighbors (KNN), Support Vector Regression (SVR, RBF kernel), Decision Tree, Random Forest, Extra Trees, Gradient Boosting, XGBoost, LightGBM, and Multi-Layer Perceptron (MLP). The models were trained on 80% of the data and validated on the remaining 20%, using a split stratified by the microorganism × resin combination so that every experimental condition was represented in both subsets. Hyperparameter optimization was performed using GridSearchCV with five-fold cross-validation. Model performance was evaluated using the coefficient of determination (R^2^), cross-validation R^2^, root mean square error (RMSE), mean absolute error (MAE), ceiling R^2^, and percentage of the ceiling. For each response variable, the model with the best predictive performance was selected. To verify that this procedure did not artificially inflate predictive performance through leakage between repeated measurements of the same experimental condition, a leave-one-replicate-group-out cross-validation (GroupKFold, k = 6, one fold per repeated-measurement position within each condition) was additionally performed; results are reported in the Results section.

#### 3.7.4. Interpretation of the Models

The relative importance of the experimental variables was estimated using permutation importance, based on the model selected for each response variable. The analysis was performed on the test set using 30 permutations per variable. Subsequently, the individual contribution of each category was evaluated using SHAP (SHapley Additive exPlanations) values, reporting the mean absolute SHAP value and the mean SHAP effect for each category. Finally, the effect of treatment concentration was evaluated using Partial Dependence Plots constructed from the models selected for each response variable. All machine learning analyses were performed in Python (version 3.10; Python Software Foundation) using the Scikit-learn 1.8.0, XGBoost 3.3.0, LightGBM 4.7.0, and SHAP 0.52.0.

### 3.8. Use of Generative AI-Assisted Tools in Manuscript Preparation

During the preparation and review of this manuscript, ChatGPT 5.0 (OpenAI) was used as an assistive tool to support the organization and linguistic refinement of selected sections of the text and to improve clarity and scientific writing. This AI tool was not used to generate or modify experimental data, conduct laboratory experiments, or independently establish the scientific conclusions of the study. All AI-assisted content was critically reviewed, verified, and edited by the authors, who retain full responsibility for the accuracy, interpretation, and final content of the manuscript.

## 4. Conclusions

This study demonstrates that *Cannabis sativa* resins exhibit antibacterial and antifungal activity, as well as activity against pre-formed biofilms, with the magnitude and pattern of the response varying according to microorganism identity, treatment concentration, resin identity, and the biological endpoint evaluated. Planktonic growth inhibition showed a clearer concentration-dependent pattern, whereas reduction in pre-formed biofilm biomass was more heterogeneous, highlighting the distinct behavior of these two biological responses. Inferential analysis showed that microorganism identity and treatment concentration were significantly associated with both endpoints, while the contribution of resin identity was phenotype-dependent, being more evident for the pre-formed biofilm response than for planktonic growth inhibition. Consistently, interpretable machine-learning analyses identified microorganism identity as the variable providing the greatest predictive information, followed by treatment concentration, whereas resin identity made a comparatively smaller contribution to model predictions. SHAP and Partial Dependence analyses further supported the existence of endpoint-specific response patterns and showed that the predictive contribution of individual microorganism and resin categories varied between planktonic growth and pre-formed biofilm biomass reduction.

Overall, the integration of phytochemical characterization, microbiological phenotyping, inferential statistics, and interpretable machine learning provides a robust analytical framework for investigating complex natural-product matrices and highlights the importance of considering microbial identity and biological phenotype when interpreting their antimicrobial activity.

## Figures and Tables

**Figure 1:**
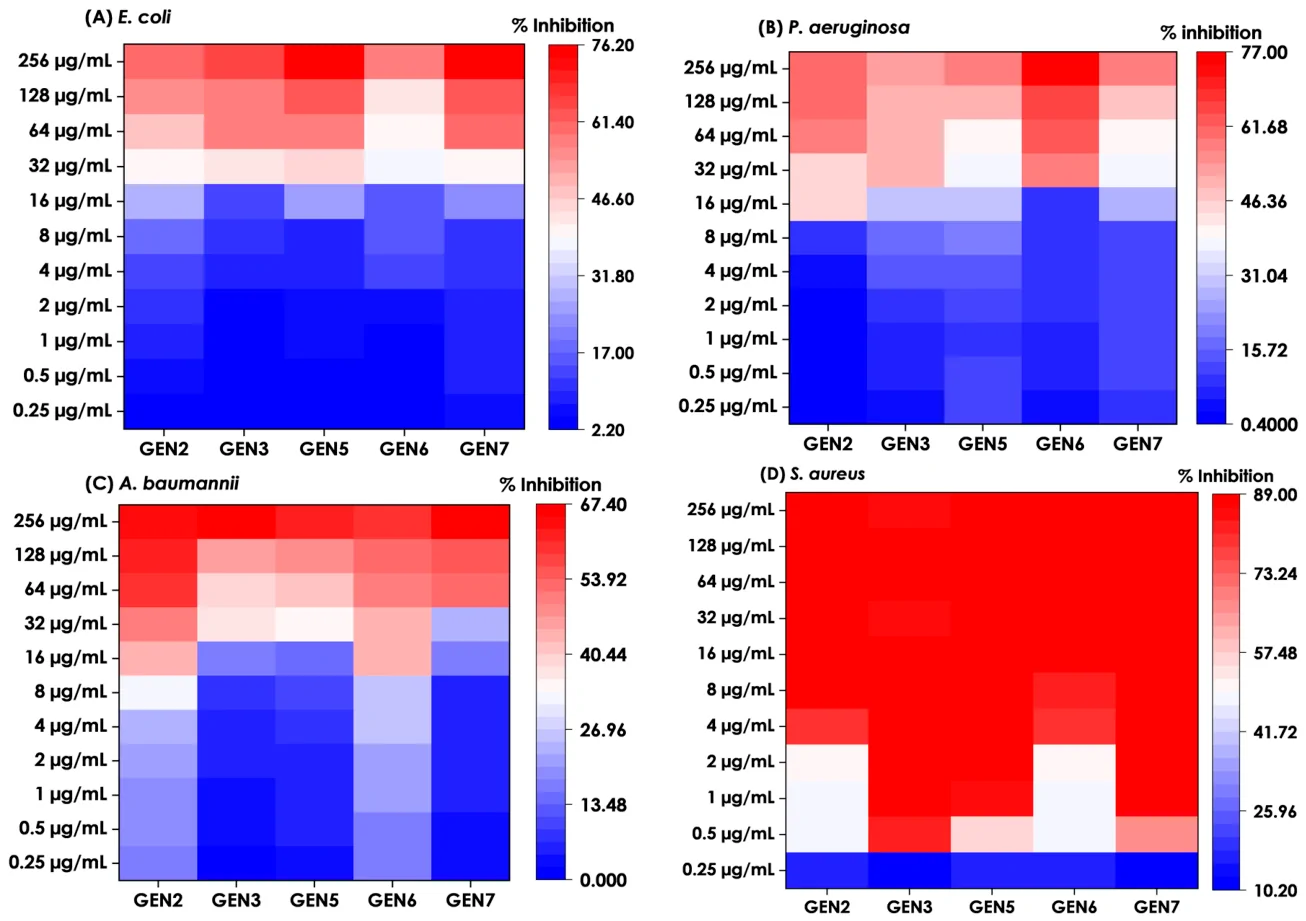
Concentration-dependent antibacterial inhibition profiles of *Cannabis sativa* resins visualized using a heat map. The heat map shows the average percentages of bacterial inhibition obtained for each resin against each tested bacterium at different concentrations. The color intensity indicates the level of bacterial inhibition, ranging from low (blue) to high (red).

**Figure 2:**
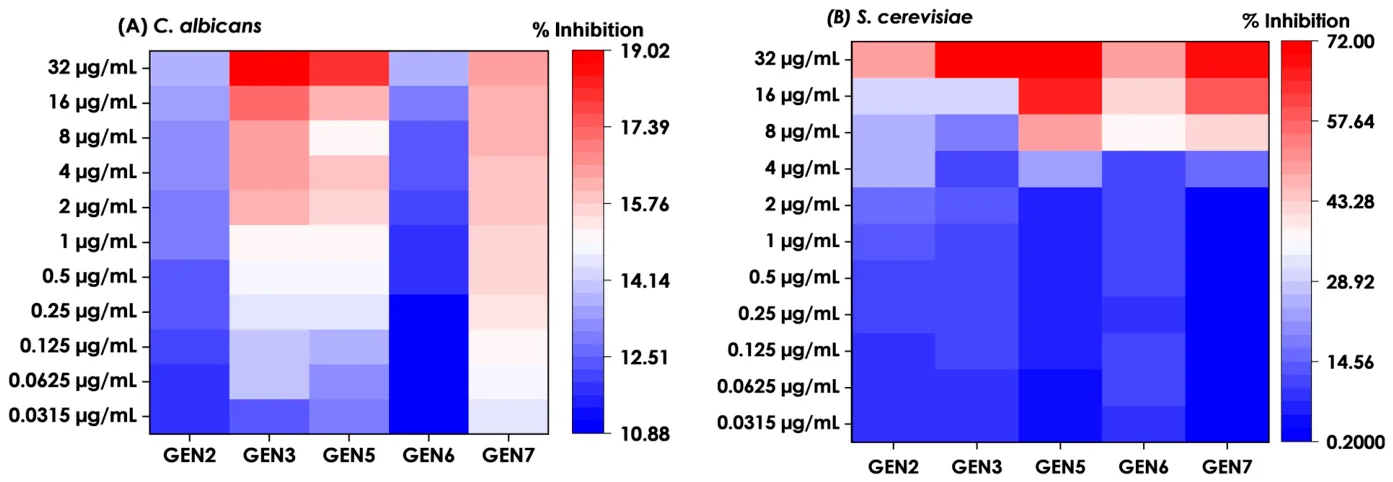
Inhibition profiles in yeasts dependent on the concentration of *Cannabis sativa* resins, visualized using a heatmap. The heatmap represents the average percentages of inhibition obtained for each resin against each yeast evaluated at different concentrations. The color intensity indicates the level of bacterial inhibition, ranging from low (blue) to high (red).

**Figure 3:**
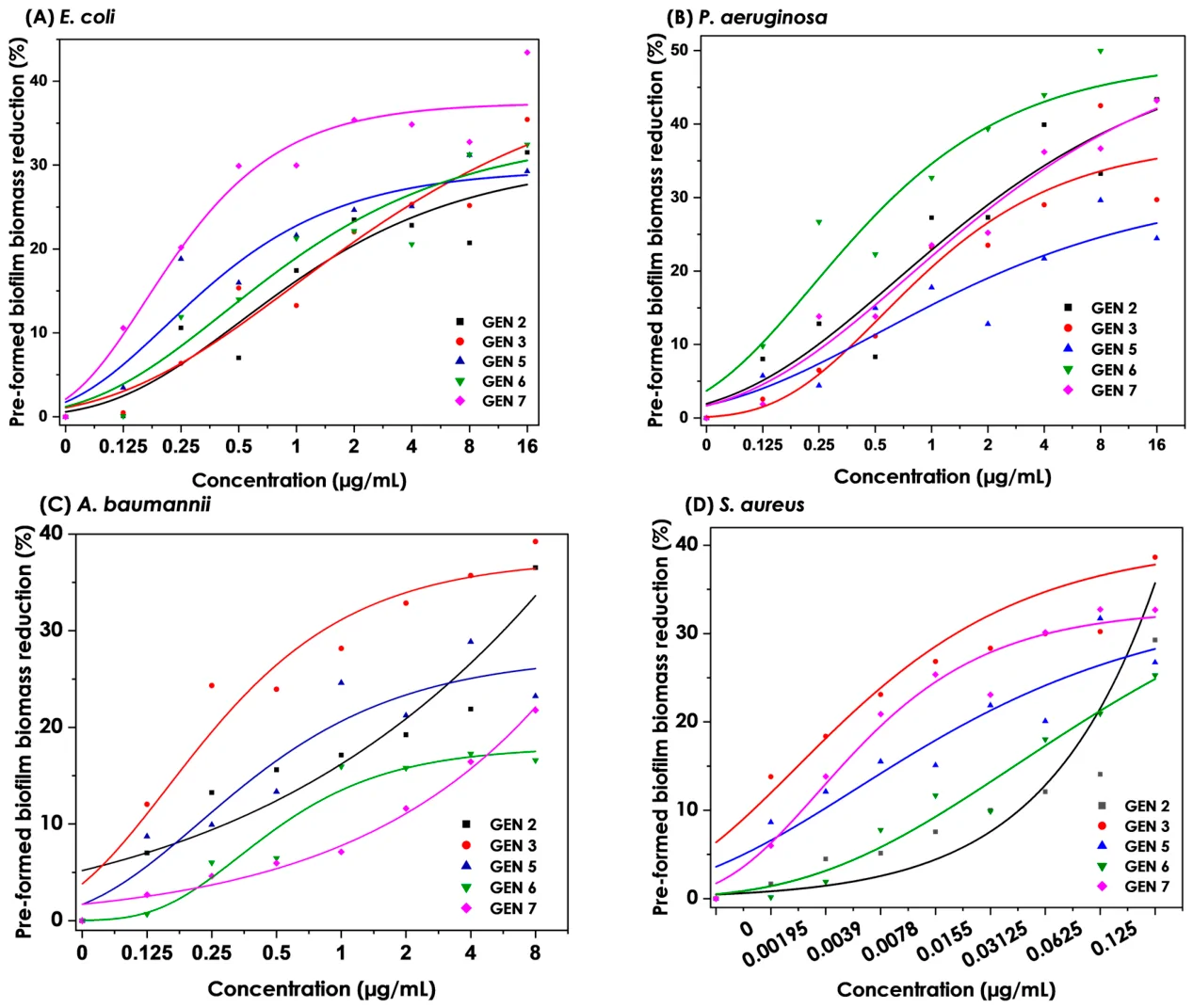
Gompertz model fits for the concentration-dependent reduction in pre-formed biofilm biomass by *Cannabis sativa* resins against Gram-negative and Gram-positive bacteria. Symbols represent experimental observations, whereas solid lines correspond to the fitted Gompertz models. Pre-formed biofilm biomass reduction is expressed as a percentage relative to untreated biofilm control.

**Figure 4:**
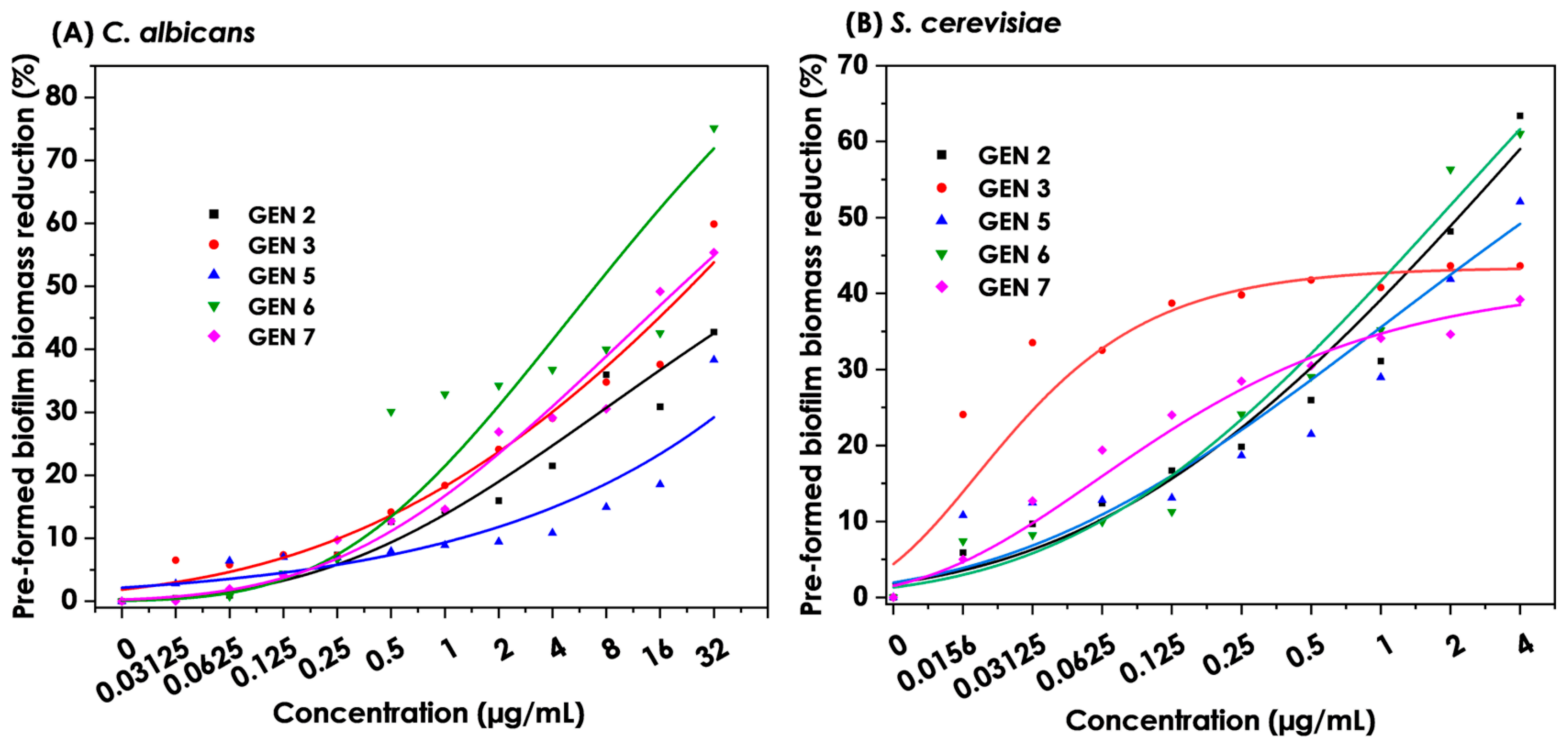
Gompertz model fits for the concentration-dependent reduction in pre-formed biofilm biomass by *Cannabis sativa* resins against *C. albicans* and *S. cerevisiae*. Symbols represent experimental observations, whereas solid lines correspond to the fitted Gompertz models. Pre-formed biofilm biomass reduction is expressed as a percentage relative to untreated biofilm control.

**Figure 5:**
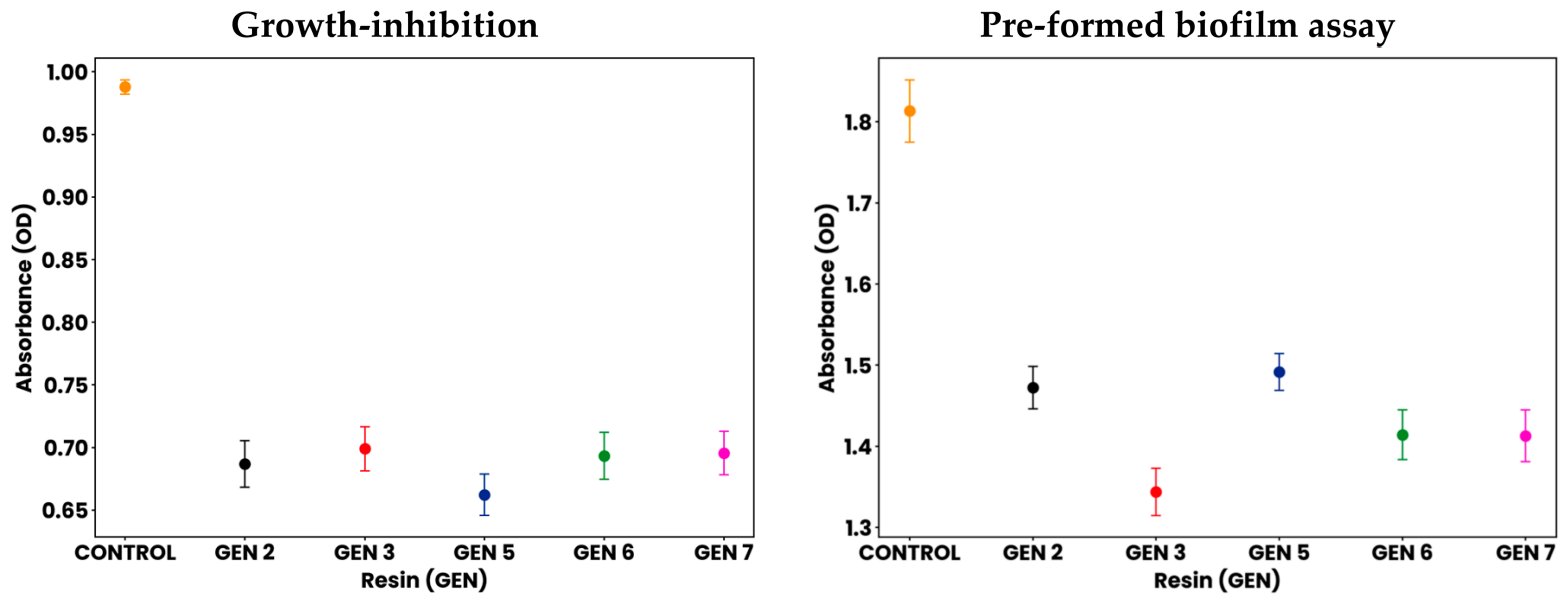
Mean absorbance (±95% CI) by resin treatment: growth inhibition and pre-formed biofilm assays.

**Figure 6:**
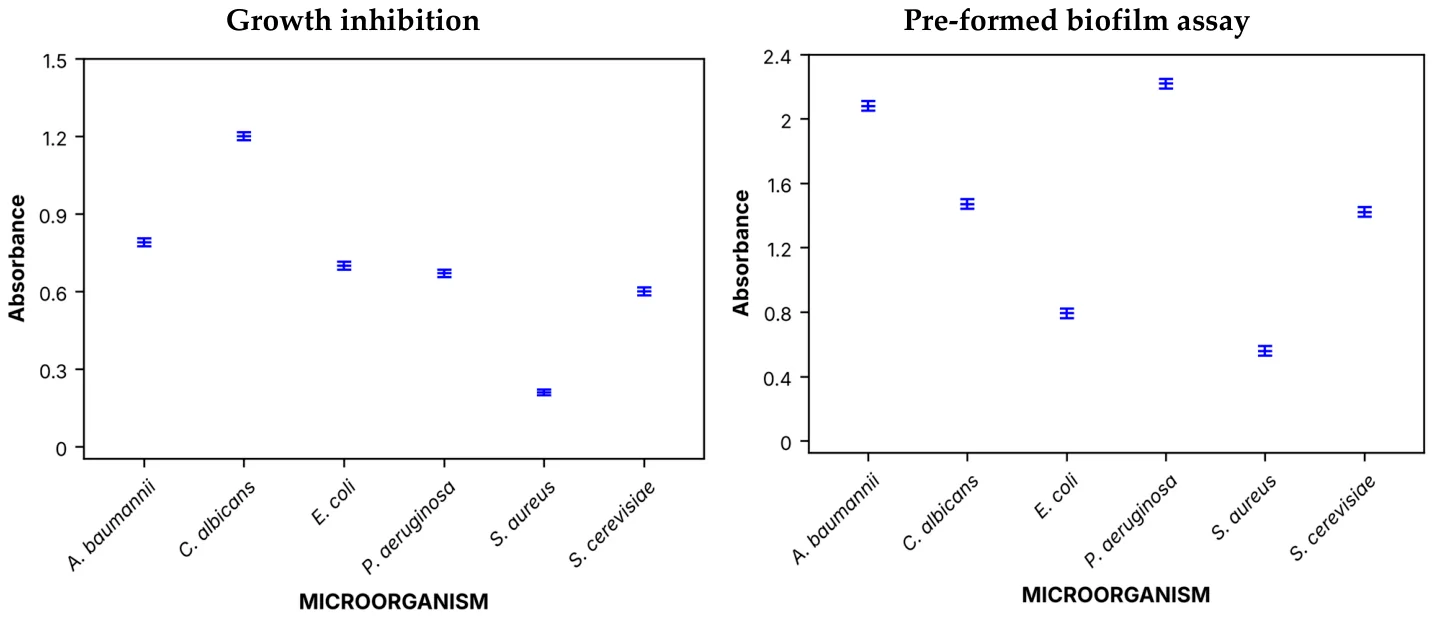
Mean absorbance (±95% CI) by resin treatment: growth inhibition and pre-formed biofilm assays.

**Figure 7:**
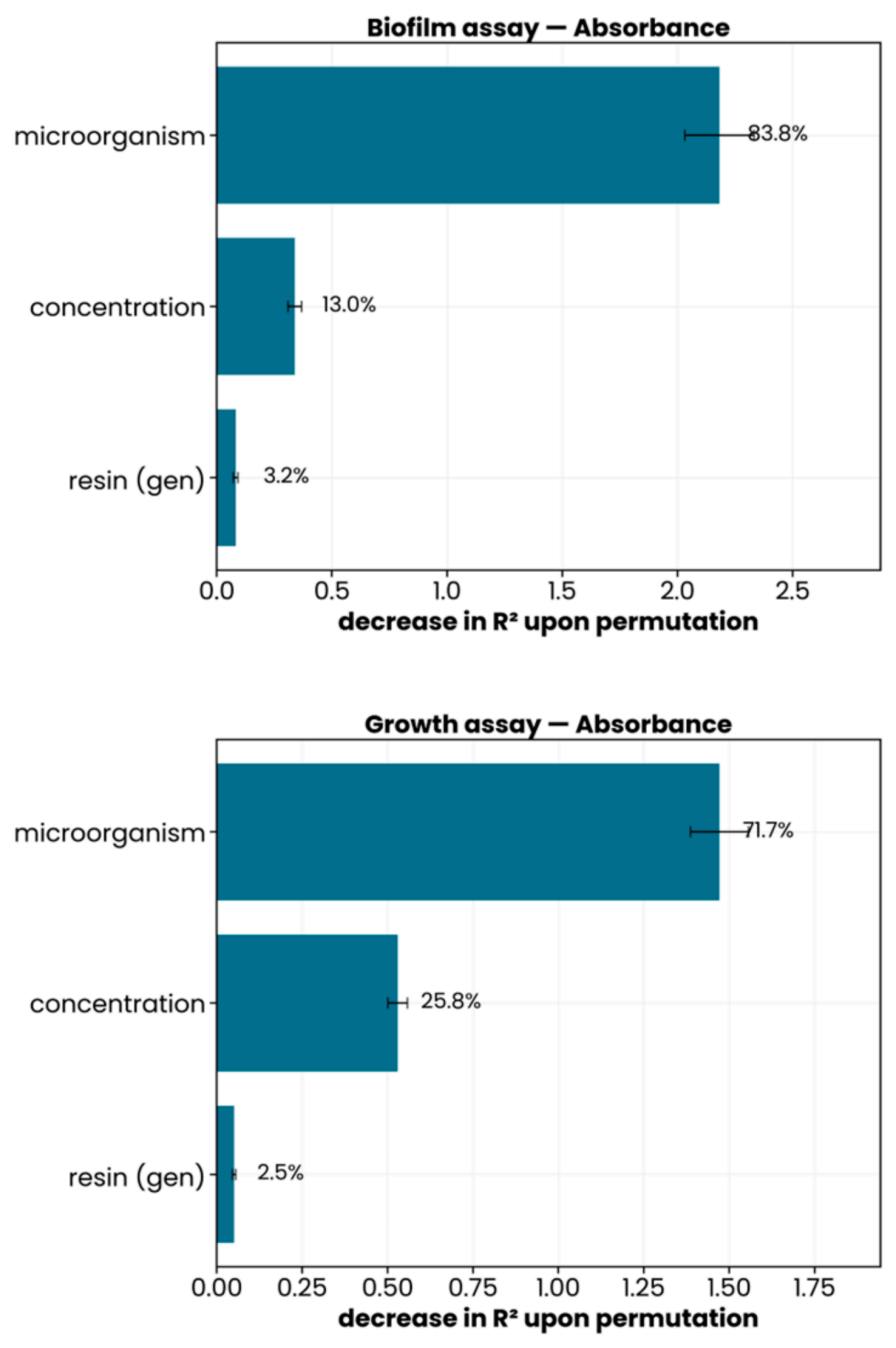
Relative importance of microorganism identity, treatment concentration, and resin identity estimated by permutation importance analysis for absorbance responses during growth inhibition and pre-formed biofilm biomass reduction.

**Figure 8:**
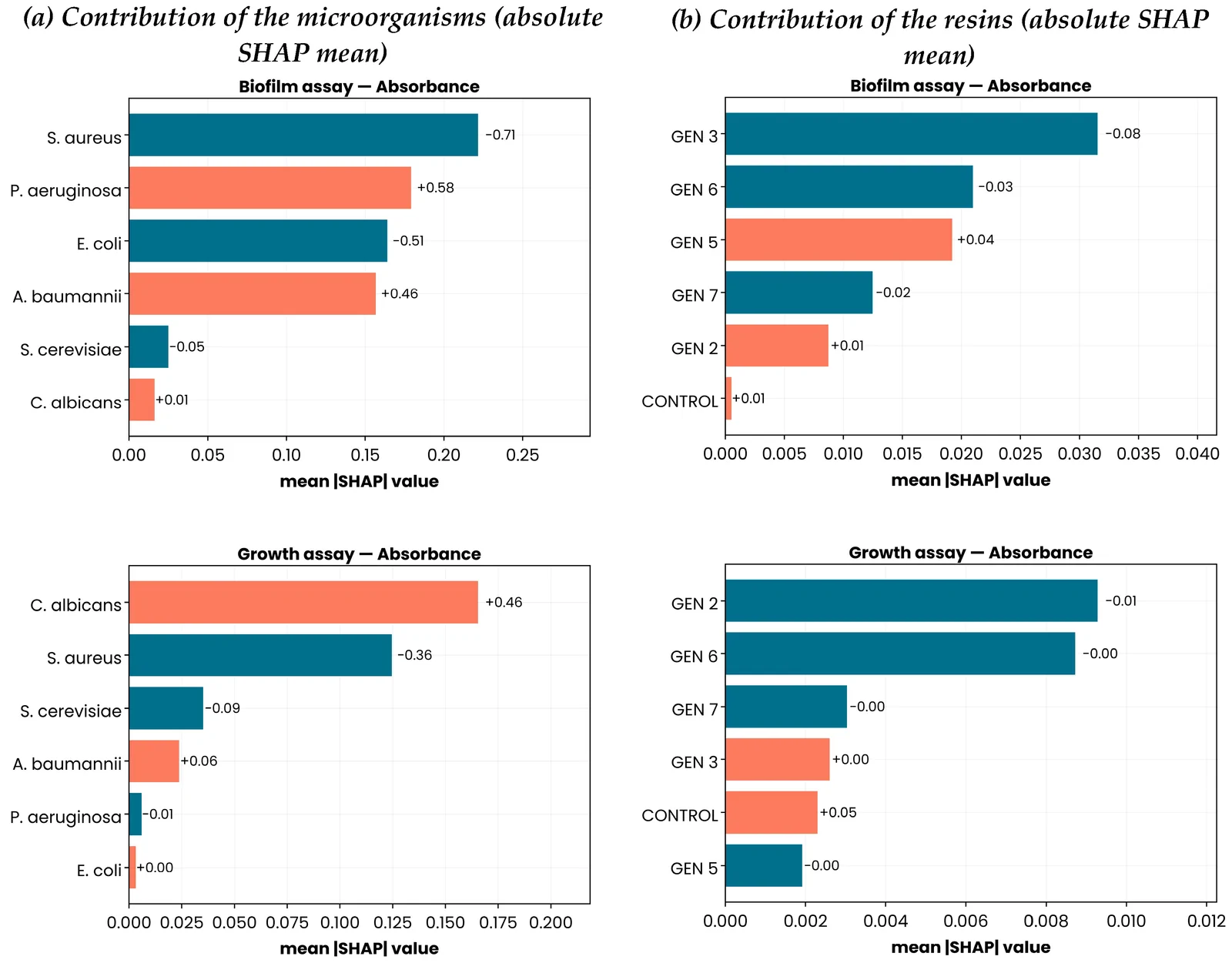

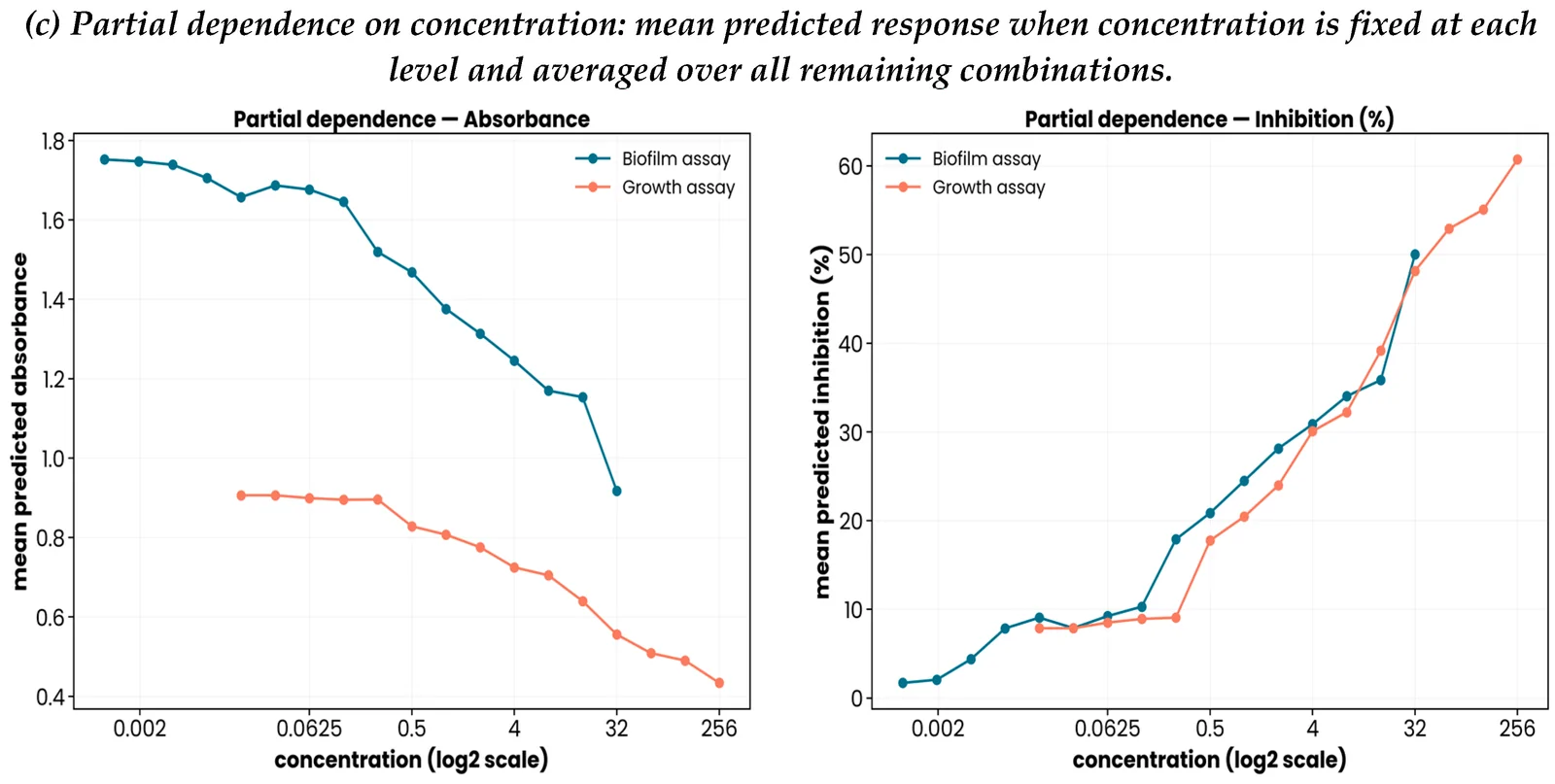
SHAP interpretation and partial dependence analysis of microorganism identity, resin identity, and treatment concentration for growth inhibition and pre-formed biofilm biomass reduction.

**Figure 9:**
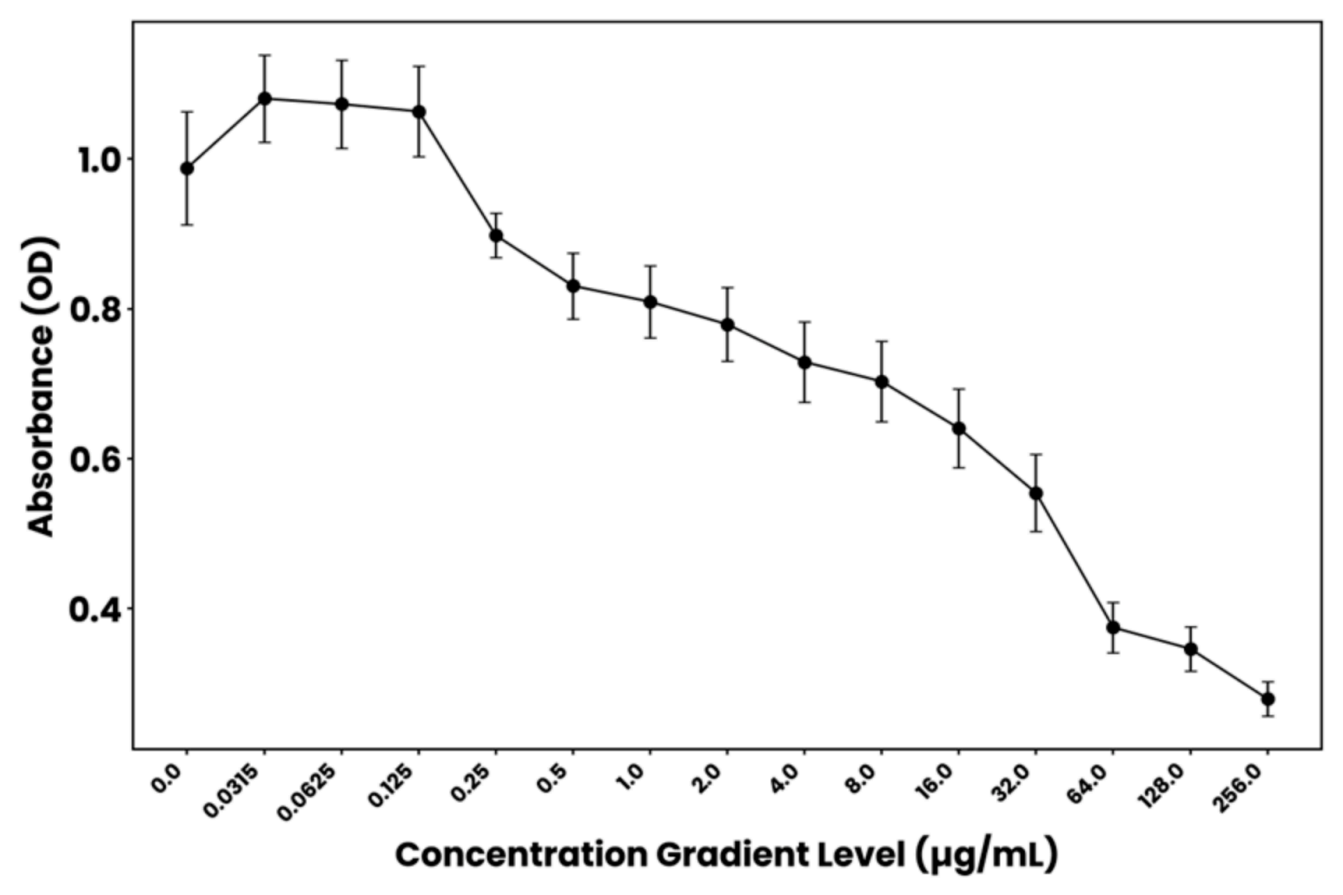
Descriptive changes in mean absorbance (±95% CI) across the concentration gradient during the growth-inhibition assay.

**Table 1:** Chemical composition of the cannabis resins GEN 2, 3, 5, 6 and 7.

Analysis	Resins				
	GEN 2	GEN 3	GEN 5	GEN 6	GEN 7
**Cannabinoids (g/100 g, wet basis)**					
d9-Tetrahydrocannabinoid (Δ^9^-THC)	2.85	2.3	2.16	3.19	2.2
Cannabidiol (CBD)	80.97	40.01	27.99	50.32	34.20
Cannabidiolic acid (CBDA)	1.61	39.16	51.06	34.21	44.83
Cannabidivarin (CBDV)	0.11	0.06	0.06	0.12	0.06
Cannabigerol (CBG)	0.95	0.32	0.87	1.30	0.45
Cannabichromene (CBC)	3.07	1.58	1.35	2	1.31
**Terpenes (mg/kg)**					
β-Myrcene	394.79	412.90	683.83	593.34	978.27
Guaiol	3304.96	2038.28	2969.61	4821.02	4132.38
β -Pinene	12.89	28.10	42.83	39.15	67.60
d-3-Carene	15.96	17.60	22.43	18.95	19.03
Ocimene	248.44	190.79	190.07	112.34	156.59
Limonene	114.28	112.79	165.55	181.28	179.69
α-Bisabolol	2812.05	1360.90	1432.41	1604.94	1658.45
Nerolidol 1	428.14	210.46	310.08	438.71	317.16
a-Humulene	1472.68	758.27	861.13	857.76	1134.17
γ-Terpinene	16.49	14.59	14.48	16.33	14.95
Terpinolene	20.76	21.98	19.34	21.19	18.43
Linalool	341.54	161.05	480.26	630.42	236.23
β -Caryophyllene	4178.75	2097.38	2324.42	2498.19	3168.5
Caryophyllene oxide	727.37	345.55	375.37	579.4	637.85
Cineole (Eucalyptol)	50.63	39.03	26.13	26.48	40.76

**Table 2:** Minimum inhibitory concentrations (MICs) of *Cannabis sativa* resins and reference antimicrobial agents against the tested microorganisms. MIC values are expressed in µg/mL. Values preceded by “>” indicate that complete growth inhibition was not achieved at the highest concentration tested. MBC and MFC values were not determined in this study.

Microorganism	Gentamicin	Ketoconazole	GEN2	GEN3	GEN5	GEN6	GEN7
*E. coli*	1.0	-	>256.0	>256.0	>256.0	>256.0	>256.0
*P. aeruginosa*	1.0	-	>256.0	>256.0	>256.0	>256.0	>256.0
*A. baumannii*	16.0	-	>256.0	>256.0	>256.0	>256.0	>256.0
*S. aureus*	1.0	-	8.0	1.0	2.0	8.0	2.0
*C. albicans*	-	>32	>32	>32	>32	>32	>32
*S. cerevisiae*	-	0.125	>32	>32	>32	>32	>32

**Table 3:** Absorbance by microorganism species in the growth inhibition and pre-formed biofilm assays.

Microorganism	Assay	Control Mean	Treated Mean (GEN2–7)	Overall Mean	Reduction (%)
***E. coli***	Growth-inhibition	0.905	0.671	0.710	25.8%
	Pre-formed biofilm assay	0.961	0.763	0.796	20.7%
***A. baumannii***	Growth-inhibition	1.056	0.766	0.814	27.4%
	Pre-formed biofilm assay	2.534	2.054	2.134	18.9%
***S. aureus***	Growth-inhibition	0.740	0.181	0.274	75.6%
	Pre-formed biofilm assay	0.788	0.642	0.667	18.5%
***C. albicans***	Growth-inhibition	1.454	1.248	1.282	14.1%
	Pre-formed biofilm assay	1.744	1.399	1.456	19.8%
***S. cerevisiae***	Growth-inhibition	0.886	0.609	0.655	31.3%
	Pre-formed biofilm assay	1.962	1.495	1.573	23.8%

**Table 4:** Summary of generalized linear model (GLM) analyses for absorbance responses during growth inhibition and pre-formed biofilm assays.

Assay	Factor	df	F	*p*-Value	Significance
**Growth inhibition**	Microorganism	5	1673.49	<0.0001	***
	Resin identity	4	0.43	0.7889	ns
	Concentration	1	1397.36	<0.0001	***
**Pre-formed biofilm**	Microorganism	5	1970.69	<0.0001	***
	Resin identity	4	21.79	<0.0001	***
	Concentration	1	714.68	<0.0001	***

*** = significant relationship; ns = non-significant relationship.

**Table 5:** Predictive performance of machine learning models for absorbance responses during growth inhibition and pre-formed biofilm assays.

Model	Growth Inhibition R^2^CV	Growth Inhibition Test R^2^	Growth Inhibition RMSE	Pre-Formed Biofilm R^2^CV	Pre-Formed Biofilm Test R^2^	Pre-Formed Biofilms
**XGBoost**	0.9922	0.9918	0.0327	0.9591	0.9440	0.1447
**Random Forest**	0.9921	0.9920	0.0322	0.9585	0.9437	0.1450
**LightGBM**	0.9921	0.9918	0.0326	0.9595	0.9459	0.1423
**KNN**	0.9920	0.9917	0.0328	0.9560	0.9423	0.1469
**Decision Tree**	0.9920	0.9917	0.0328	0.9559	0.9423	0.1469
**Extra Trees**	0.9920	0.9917	0.0328	0.9588	0.9443	0.1443
**Gradient Boosting**	0.9909	0.9899	0.0363	0.9595	0.9438	0.1450
**SVR-RBF**	0.9882	0.9872	0.0409	0.9518	0.9411	0.1484
**Neural Network (MLP)**	0.9754	0.9766	0.0552	0.9499	0.9371	0.1533
**Ridge Regression**	0.8999	0.8997	0.1143	0.8990	0.8897	0.2031

**Bold:** Best performance for each metric.

## Data Availability

The original contributions presented in this study are included in the article and Supplementary Materials. Further inquiries can be directed to the corresponding author (C.M.).

## Supplementary Materials

The following supporting information can be downloaded at: https://www.mdpi.com/article/10.3390/molecules31214028/s1. Table S1. Inhibition percentages of bacterial strains in the presence of different concentrations of cannabis resins; Table S2. Yeast inhibition percentages in the presence of different concentrations of cannabis resins; Table S3. Percentages of pre-formed biofilm biomass reduction for each bacterial strain in the presence of different concentrations of *Cannabis sativa* resins; Table S4. Percentages of pre-formed biofilm biomass reduction for each yeast strain in the presence of different concentrations of *Cannabis sativa* resins; Table S5. Machine learning algorithms and hyperparameters evaluated during the optimization process; Table S6. Results of the Permutation Importance analysis for the selected machine learning models; Table S7. Pre-formed biofilm assay—Absorbance; Table S8. Pre-formed biofilm assay—% inhibition; Table S9. Growth—Absorbance; Table S10. Growth—% inhibition; Table S11. Pre-formed biofilm assay—Absorbance; Table S12. Pre-formed biofilm assay—% inhibition; Table S13. Growth—Absorbance; Table S14. Growth—% inhibition; Table S15. Pre-formed biofilm % assay; Table S16. Growth—% inhibition; Table S17. Botanical identification, regulatory registration, geographical origin, and cultivation characteristics of the *Cannabis sativa* L. cultivars used to produce the evaluated resins; Figure S1. Cross-validation R^2^ of the ten model families optimized with GridSearchCV; Figure S2. Observed vs. predicted values in the test set (20% of the data, not used in training or hyperparameter selection); Figure S3. Importance by permutation of the three input variables in each case; Figure S4. Partial concentration dependence: mean predicted response by fixing the concentration at each level and averaging over all other combinations; Figure S5. HPLC-PDA chromatogram of cannabinoid profile (a) CBDV, CBG, and CBC, (b) CBDA and Δ^9^-THC and (c) cannabidiol (CBD) in the diluted resin sample for GEN2; Figure S6. HPLC-PDA chromatogram of cannabinoid profile (a) CBDV, CBG, and CBC, (b) CBDA and Δ^9^-THC and (c) cannabidiol (CBD) in the diluted resin sample for GEN3; Figure S7. HPLC-PDA chromatogram of cannabinoid profile (a) CBDV, CBG, and CBC, (b) CBDA and Δ^9^-THC and (c) cannabidiol (CBD) in the diluted resin sample for GEN5; Figure S8. HPLC-PDA chromatogram of cannabinoid profile (a) CBDV, CBG, and CBC, (b) CBDA and Δ^9^-THC and (c) cannabidiol (CBD) in the diluted resin sample for GEN6; Figure S9. HPLC-PDA chromatogram of cannabinoid profile (a) CBDV, CBG, and CBC, (b) CBDA and Δ^9^-THC and (c) cannabidiol (CBD) in the diluted resin sample for GEN7.

## Abbreviations

ANOVA	Analysis of Variance
ATCC	American Type Culture Collection
CBC	Cannabichromene
CBD	Cannabidiol
CBDA	Cannabidiolic acid
CBDV	Cannabidivarin
CBG	Cannabigerol
DNA	Deoxyribonucleic Acid
HPLC	High-Performance Liquid Chromatography
HPLC-PDA	High-Performance Liquid Chromatography with Photodiode Array Detection
LOQ	Limit of quantification
MIC	Minimum Inhibitory Concentration
MRSA	Methicillin-Resistant *Staphylococcus aureus*
SD	Standard Deviation
THC	Δ9-Tetrahydrocannabinol
DMSO	Dimethyl Sulfoxide
OD	Optical Density
PBS	Phosphate-Buffered Saline
GLMs	Generalized linear models
