# Supplementary material for: From Phytochemical Complexity to Biological Response: Explainable Modeling of the Antimicrobial Activity of Cannabis sativa Resin
Source: Molecules. 2026 Nov 9;31(21):4028. doi: 10.3390/molecules31214028 (PMC13764897; doi:10.3390/molecules31214028)
Supplement: Supplementary file 1 [file molecules-31-04028-s001.zip › molecules-4572281-supplementary.pdf]

FROM PHYTOCHEMICAL COMPLEXITY TO BIOLOGICAL RESPONSE: EXPLAINABLE MODELING OF THE ANTIMICROBIAL ACTIVITY OF *CANNABIS SATIVA* RESIN

Dayanna Orosco[1], Andrea Ramos-Hernández, [2] Mario Romero,[2] María L. Ospina-Castro, [2] Victoria Arana, [3] Jolián Andrés Vargas Alzate,[4] Alexander Parody, [5] Carlos Mario Meléndez Gómez[1]*.

[1] Grupo de Investigación en Química Orgánica y Biomedicina, Programa de Química, Facultad de Ciencias Básicas, Laboratorio de Investigación en Biología Molecular, Universidad del Atlántico, Carrera 30 No.8-49, Puerto Colombia 081007, Colombia.
[2] Grupo de Investigación Química Supramolecular Aplicada, Programa de Química, Facultad de Ciencias Básicas, Universidad del Atlántico, Carrera 30 No.8-49, Puerto Colombia 081007, Colombia.
[3] Grupo de Investigación Ciencias, Educación y Tecnología–CETIC, Facultad de Ciencias Básicas, Universidad del Atlántico, Carrera 30 No. 8–49, Puerto Colombia 081007, Colombia.
[4]CUBIKAN GROUP SAS. Finca Veracruz, vereda La Diamantina - Km 10 vía La Argelia (Ansermanuevo Valle del Cauca), Colombia.
[5] Facultad de Ingeniería, Universidad Libre, Barranquilla, Colombia

* Corresponding author. Universidad del Atlántico, Facultad de Ciencias Básicas, Programa de Química. Carrera 30 No.8-49, Puerto Colombia 081007, Colombia. E-mail: carlosmelendez@mail.uniatlantico.edu.co

SUPPLEMENTARY INFORMATION

Section S1. Inhibition percentages

Table S1. Inhibition percentages of bacterial strains in the presence of different concentrations of cannabis resins. A value of 100 corresponds to the maximum inhibition of each strain with the treatment. Asterisks indicate statistically significant differences (*: p < 0,05; **: p < 0,02; ***: p < 0,01).

Bacteria	Concentration	Control	DMSO	Gentamicin	GEN 2	GEN 3	GEN 5	GEN 6	GEN 7
E. coli	256 µg/mL	0	2.90% ± 0.84	100	60,88 ± 2,26***	64,6 ± 1,03***	74,53 ± 1,03***	58,24 ± 3,01***	76,11 ± 0,49***
	128 µg/mL				54,79 ± 2,8***	58,53 ± 0,73***	62,24 ± 1***	43,83 ± 1,21***	61,95 ± 1,14***
	64 µg/mL				49,04 ± 1,18***	56,76 ± 0,92***	58,72 ± 0,82***	41,2 ± 1,95***	59,07 ± 0,38***

	32 µg/mL				39,88 ± 1,2***	42,98 ± 1,3***	45,06 ± 1,39***	39,08 ± 1,06***	41,39 ± 1,31***
	16 µg/mL				28,92 ± 2,34***	12,43 ± 1,38***	24,63 ± 2,33***	16,65 ± 7,11***	23,26 ± 1,72***
	8 µg/mL				17,26 ± 2,52***	10,96 ± 1,68***	8,92 ± 2,7***	15,55 ± 8,98***	11,76 ± 1,42***
	4 µg/mL				12,28 ± 1,76***	8,99 ± 2,37***	8,27 ± 2,14***	12,37 ± 7,01 ***	11,2 ± 2,46***
	2 µg/mL				11,56 ± 1,51***	4,46 ± 1,78	5,96 ± 1,53	5,59 ± 7,12	8,62 ± 1,8***
	1 µg/mL				7,29 ± 2,38***	4,22 ± 1,88	4,79 ± 1,85	3,75 ± 1,75	7,61 ± 1,4***
	0.5 µg/mL			61,3 ± 0,54***	6,8 ± 2,33**	2,84 ± 1,8	3,23 ± 2,18	2,68 ± 2,34	7,6 ± 2,73***
	0.25 µg/mL			28,6 ± 0,74***	4,37 ± 2,63	2,92 ± 2,32	3,18 ± 1,64	2,34 ± 2,83	5,91 ± 1,2
	256 µg/mL				60,08 ± 2,85***	52,01 ± 2,56***	59,08 ± 0,17***	76,83 ± 0,63***	57,67 ± 0,45***
	128 µg/mL				61,49 ± 1,12***	51,05 ± 1,49***	48,93 ± 0,51***	64,44 ± 0,85***	47,01 ± 0,39***
	64 µg/mL				57,03 ± 1,41***	49,51 ± 1,87***	41,19 ± 0,17***	63,54 ± 0,65***	39,77 ± 0,48***
	32 µg/mL				45,83 ± 1,5***	49,16 ± 1,07***	37,97 ± 0,29***	56,64 ± 0,99***	37,12 ± 0,32***
	16 µg/mL			100	44,5 ± 1,51***	29,9 ± 0,63***	29,11 ± 1,39***	9,97 ± 1,72***	26,48 ± 0,57***
P. aeruginosa	8 µg/mL	0	1.90% ± 0.04		8,66 ± 1,94***	17,25 ± 0,73***	19,02 ± 0,71***	9,97 ± 1,72***	12,57 ± 0,46***
	4 µg/mL				4,77 ± 1,46	15,18 ± 2,06***	13,31 ± 1,19***	10,24 ± 1,36***	12,45 ± 0,41***
	2 µg/mL				2,14 ± 1,34	9,54 ± 1,99***	12,77 ± 0,26***	9 ± 2,09***	11,85 ± 0,33***
	1 µg/mL				0,66 ± 1,48	7,71 ± 2,32**	10,07 ± 0,11***	7,88 ± 1,85**	11,77 ± 1,88***
	0.5 µg/mL			60,5 ± 0,28***	0,57 ± 1,6	7,33 ± 1,76**	12,25 ± 0,62***	5,68 ± 1,53	10,91 ± 1,36***

	0.25 µg/mL			27,1 ± 0,84**	0,42 ± 1,69	3,62 ± 2,1	11,95 ± 0,23***	5,33 ± 2,81	10,34 ± 0,22***
A. baumannii	256 µg/mL	0	1.24% ± 1.34	100	64,27 ± 6,13***	67,33 ± 7,97***	61,11 ± 6,01***	59,43 ± 9,32***	66,89 ± 2,61***
	128 µg/mL				60,97 ± 8,37***	46,21 ± 9,19***	49,05 ± 1,59***	53,37 ± 9,93***	55,9 ± 0,95***
	64 µg/mL				59,16 ± 12,77***	38,39 ± 11,38***	40,99 ± 2,25***	50,91 ± 9,36***	53,22 ± 4,24***
	32 µg/mL				51,5 ± 8,39***	36,08 ± 9,1***	34,37 ± 5,42***	43,82 ± 3,23***	22,96 ± 4,05***
	16 µg/mL				44,8 ± 1,47***	16,17 ± 2,16***	15,53 ± 1,85***	43,07 ± 1,56***	16,32 ± 2,14***
	8 µg/mL			87,6 ± 0,0694***	32,96 ± 3,71***	6,75 ± 4,01	9,72 ± 2,48***	24,88 ± 3,3***	6,01 ± 1,84
	4 µg/mL			50,3 ± 0,29***	23,23 ± 5,26***	5,85 ± 2,73	8,22 ± 1,73***	25,99 ± 9,99***	5,88 ± 1,71
	2 µg/mL			31,8 ± 0,36**	22,12 ± 3,57***	5,61 ± 1,32	6,57 ± 1,52	21,35 ± 2,96***	5,62 ± 1,32
	1 µg/mL			25,4 ± 0,36**	18,79 ± 0,56***	4,1 ± 2,95	6,05 ± 0,98	20,88 ± 2,43***	4,71 ± 2,46
	0.5 µg/mL			26,65 ± 0,38**	18,35 ± 8,48***	3,86 ± 2,44	5,76 ± 1,06	17,81 ± 3,96***	4,08 ± 0,71
	0.25 µg/mL			18,79 ± 0,56**	17,01 ± 3,58***	0,18 ± 0,66	4,49 ± 0,74	16,16 ± 2,89***	3,78 ± 8,19
S. aureus	256 µg/mL	0	2.00% ± 0.09	100	100	100	100	100	100
	128 µg/mL				100	100	100	100	100
	64 µg/mL				100	100	100	100	100
	32 µg/mL				100	100	100	100	100
	16 µg/mL				100	100	100	100	100
	8 µg/mL				100	100	100	100	100
	4 µg/mL				80,07 ± 2,26***	100	100	80,07 ± 2,26***	100
	2 µg/mL				51,92 ± 4,59***	100	100	51,92 ± 4,59***	100
	1 µg/mL				49,14 ± 3,5***	100	85,28 ± 0,91***	49,14 ± 3,5***	86,48 ± 0,82***

	0.5 µg/mL			75,34 ± 1,31***	48,66 ± 3,32***	82,28 ± 1,3***	55,9 ± 9,02***	48,66 ± 3,32***	67,77 ± 2,28***
	0.25 µg/mL			46,51 ± 2,81***	16,68 ± 4,94***	11,54 ± 10,56***	16,24 ± 4,44***	16,68 ± 4,94***	10,36 ± 5,11***

Table S2. Yeast inhibition percentages in the presence of different concentrations of cannabis resins. A value of 100 corresponds to the maximum inhibition of each strain with the treatment. Asterisks indicate statistically significant differences (*: $p < 0,05$; **: $p < 0,02$; ***: $p < 0,01$).

Yeast	Concentration	Control	DMSO	Ketoconazole	GEN 2	GEN 3	GEN 5	GEN 6	GEN 7
C. albicans	**32 µg/mL**	0	1,87 ± 0,935	73,0 ± 3,30 ***	13,85 ± 1,82 ***	19,01 ± 1,06 ***	18,08 ± 0,676 ***	13,62 ± 2,69 ***	16,33 ± 0,968 ***
	**16 µg/mL**	0		48,5 ± 8,34 ***	13,53 ± 2,24 ***	17,29 ± 0,51 ***	16,15 ± 0,517 ***	12,99 ± 2,5 ***	16,29 ± 1,273 ***
	**8 µg/mL**	0		46,63 ± 9,27 ***	13,21 ± 2,37 ***	16,38 ± 0,42 ***	15 ± 0,201 ***	12,33 ± 2,56 ***	16,07 ± 0,996 ***
	**4 µg/mL**	0		46,9 ± 8,37 ***	13,19 ± 2,3 ***	16,42 ± 0,84 ***	15,95 ± 0,865 ***	12,26 ± 2,91 ***	16,02 ± 0,354 ***
	**2 µg/mL**	0		46,14 ± 8,79 ***	12,89 ± 1,5 ***	16,16 ± 1,26 ***	15,66 ± 1,181 ***	12,11 ± 2,43 ***	15,97 ± 0,335 ***
	**1 µg/mL**	0		47,71 ± 9,44 ***	12,82 ± 2,08 ***	15,19 ± 0,71 ***	15,05 ± 0,494 ***	11,8 ± 2,7 ***	15,75 ± 0,28 ***
	**0,5 µg/mL**	0		46,0 ± 9,12 ***	12,45 ± 3,08 ***	14,76 ± 0,45 ***	14,94 ± 0,923 ***	11,7 ± 2,12 ***	15,51 ± 0,598 ***
	**0,25 µg/mL**	0		47,2 ± 9,24 ***	12,43 ± 2,08 ***	14,54 ± 0,54 ***	14,58 ± 0,604 ***	11,1 ± 2,73 ***	15,4 ± 0,55 ***
	**0,125 µg/mL**	0		47,18 ± 10,52 ***	12 ± 1,76 ***	14,09 ± 0,52 ***	13,78 ± 0,62 ***	11,02 ± 2,37 ***	15,06 ± 0,43 ***
	**0,0625 µg/mL**	0		41,68 ± 9,87 ***	11,78 ± 2,11 ***	14,07 ± 0,45 ***	13,09 ± 0,522 ***	10,96 ± 2,79 ***	14,75 ± 0,518 ***
	**0,0315 µg/mL**	0		38,74 ± 1,48 ***	11,71 ± 2,28 ***	12,5 ± 2,94 ***	13,05 ± 1,034 ***	10,88 ± 2,72 ***	14,53 ± 0,458 ***
S. cerevisiae	**32 µg/mL**	0	1,61 ± 1,23	100	48,92 ± 2,19 ***	70,98 ± 0,53 ***	71,94 ± 0,29 ***	48,27 ± 2,09 ***	68,78 ± 0,4 ***
	**16 µg/mL**	0			29,78 ± 1,52 ***	29,36 ± 0,31 ***	66,45 ± 2,29 ***	42,25 ± 1,02 ***	57,72 ± 0,27 ***

	**8 µg/mL**	0			25,02 ± 1,52 ***	18,69 ± 1,25 ***	49,13 ± 3,15 ***	38,45 ± 0,44 ***	42,63 ± 2,12 ***
	**4 µg/mL**	0			24,17 ± 1,86 ***	11,98 ± 0,48 ***	23,21 ± 4,45 ***	10,56 ± 0,9 ***	16,43 ± 1,05 ***
	**2 µg/mL**	0			16,72 ± 1,83 ***	12,21 ± 4,05 ***	7,34 ± 4,07 ***	10,19 ± 1,07 ***	2,38 ± 0,92
	**1 µg/mL**	0			13,03 ± 2,53 ***	11,75 ± 3,66 ***	6,89 ± 3,68 ***	10,48 ± 1,56 ***	2,4 ± 1,53
	**0,5 µg/mL**	0			9,99 ± 1,45 **	11,4 ± 5,47 **	6,06 ± 5,53	9,79 ± 1,26 ***	0,89 ± 0,86
	**0,25 µg/mL**	0			9,81 ± 1,51 **	10,92 ± 5,5 **	6,54 ± 5,5	9,67 ± 1,27 ***	2,5 ± 0,72
	**0,125 µg/mL**	0			9,51 ± 1,72 **	10,5 ± 4,79 **	5,64 ± 4,81	9,83 ± 1,11 ***	1,19 ± 1,62
	**0,0625 µg/mL**	0		60,55 ± 0,28***	9,27 ± 1,46 **	9,39 ± 4,58 **	4,53 ± 4,61	9,81 ± 1,2 ***	0,45 ± 0,88
	**0,0315 µg/mL**	0		27,15 ± 0,84 **	9,2 ± 1,48 **	9,07 ± 3,85 **	4,21 ± 3,88	9,45 ± 1,22 ***	0,31 ± 0,94

Table S3. Percentages of pre-formed biofilm biomass reduction for each bacterial strain in the presence of different concentrations of *Cannabis sativa* resins. A value of 100 corresponds to the maximum reduction of pre-formed biofilm biomass for each strain under the treatment. Asterisks indicate statistically significant differences (*: p < 0,05; **: p < 0,02; ***: p < 0,01).

Bacteria	Concentration	Control	DMSO	GEN 2	GEN 3	GEN 5	GEN 6	GEN 7
E. coli	**16 µg/mL**	100	99.90% ± 0.04	68,91 ± 12,33 ***	64,66 ± 8,19 ***	70,98 ± 8,34***	68,15 ± 11,82***	56,8 ± 9,78***
	**8 µg/mL**			79,66 ± 10,49 ***	74,99 ± 5,23 ***	69,11 ± 5,61***	69,23 ± 9,72***	67,32 ± 7,73***
	**4 µg/mL**			77,96 ± 14,83 ***	74,88 ± 8,47 ***	75,27 ± 10,8***	79,84 ± 7,57***	65,25 ± 6***
	**2 µg/mL**			76,71 ± 11,21 ***	78,01 ± 8,21 ***	75,65 ± 10,56***	78,09 ± 9,07***	64,81 ± 9,11***
	**1 µg/mL**			83,32 ± 13,79 **	87,25 ± 11,75 **	78,46 ± 3,97***	79,37 ± 13,22***	70,37 ± 9,81***
	**0,5 µg/mL**			93,17 ± 7,32	84,87 ± 7,68 **	84,32 ± 7,84***	78,87 ± 19,09***	70 ± 13,75***
	**0,25 µg/mL**			89,58 ± 14,1	94,04 ± 7,59	81,26 ± 12,46***	88,6 ± 9,62***	79,75 ± 14,9***
	**0,125 µg/mL**			112,09 ± 12,28	100,03 ± 9,69	96,85 ± 11,59	96,7 ± 13,82 *	89,69 ± 7,58 **
P. aeruginosa	**16 µg/mL**	100	98.95% ± 0.04	55,81 ± 9,78 ***	70,11 ± 5,69 ***	74,56 ± 12,02 ***	55,89 ± 10,13 ***	56,91 ± 6,99 ***
	**8 µg/mL**			65,75 ± 11,48 ***	56,84 ± 8,6 ***	69,85 ± 7,52 ***	49,71 ± 5,71 ***	62,92 ± 5,69 ***
	**4 µg/mL**			60,72 ± 2,94 ***	71,33 ± 1,82 ***	77,05 ± 14,15 ***	56,65 ± 1,73 ***	64,89 ± 5,25 ***
	**2 µg/mL**			73,36 ± 0,28 ***	76,53 ± 4,47 ***	87,91 ± 6,52 ***	60,76 ± 3,43 ***	73,98 ± 5,65 ***
	**1 µg/mL**			73,48 ± 0,56 ***	75,67 ± 12,42 ***	81,71 ± 9,16 ***	66,48 ± 10,52 ***	77,06 ± 8,92 ***
	**0,5 µg/mL**			91,07 ± 10,46	88,82 ± 6,14 *	84,61 ± 8,95 **	77,45 ± 7,18 ***	86,3 ± 4,78 ***
	**0,25 µg/mL**			87,2 ± 5,46	93,51 ± 5,9	94,52 ± 13,94	73,64 ± 2,75 ***	86,3 ± 4,78 ***
	**0,125 µg/mL**			93,04 ± 1,86	103,27 ± 2,69	93,81 ± 9,62	90,7 ± 2,66 **	103,28 ± 9,02 **

	**32 µg/mL**			--	--	--	--	67,69 ± 3,07 ***
	**16 µg/mL**			76,57 ± 9,74 ***	57,26 ± 6,55 ***	65,05 ± 8,34 ***	--	73,45 ± 2,57 ***
	**8 µg/mL**			59,9 ± 6,82 ***	59,88 ± 8,84 ***	75,68 ± 3,36 ***	84,23 ± 5,7 ***	78,01 ± 13,25 ***
A. baumannii	**4 µg/mL**	100	99.24% ± 1.34	75,34 ± 7,64 ***	64,95 ± 12,51 ***	70,02 ± 5,53 ***	82,71 ± 5,15 ***	83,39 ± 6,67 ***
	**2 µg/mL**			80,1 ± 9,73 ***	67 ± 11,69 ***	78,01 ± 4,12 ***	84,27 ± 4,46 ***	89,26 ± 4,89 **
	**1 µg/mL**			84,05 ± 11,73 ***	69,98 ± 6,61 ***	74,79 ± 9,26 ***	83,87 ± 5,39 ***	91,53 ± 4,25
	**0,5 µg/mL**			80,9 ± 9,35 ***	73,65 ± 8,46 ***	85,26 ± 7,43 ***	93,3 ± 11,25	96,64 ± 5,26
	**0,25 µg/mL**			78,58 ± 8,4 ***	75,43 ± 8,51 ***	89,91 ± 10,27 **	93,91 ± 6,07	95,24 ± 5,38
	**0,125 µg/mL**			89,36 ± 11,51 **	86,04 ± 9,87 *	88,56 ± 10,67 *	99,31 ± 6,7	95,06 ± 5,27
	0,25 µg/mL			61,52 ± 9,75 ***	52,24 ± 10,93 ***	74,32 ± 10,44 ***	75,95 ± 12,02 ***	68,25 ± 9,15 ***
	0,125 µg/mL			86,49 ± 8,45 *	70,75 ± 9,61 ***	69,18 ± 9,07 ***	83,24 ± 12,33 ***	68,22 ± 9,41 ***
	0,0625 µg/mL			89,06 ± 11,73	71,02 ± 9,77 ***	81,06 ± 12,68 ***	80,46 ± 14,18 ***	70,83 ± 9,59 ***
	0,03125 µg/mL			100,7 ± 16,84	72,51 ± 13,47 ***	89,21 ± 13,18 ***	91,57 ± 14,87	77,88 ± 9,58 ***
S. aureus	0,0155 µg/mL	100	98.00% ± 0.09	97,34 ± 18,8	77,64 ± 9,93 ***	79,22 ± 10,69 ***	89,92 ± 15,96	75,71 ± 10,59 ***
	0,0078 µg/mL			93,32 ± 10,22	75,08 ± 12 ***	85,66 ± 11,58 ***	93,68 ± 18,72	80,64 ± 17,69 ***
	0,00390 µg/mL			96,27 ± 15	73,45 ± 13,98 ***	92,96 ± 16,75 ***	99,42 ± 12,79	86,87 ± 12,73 ***
	0,00195 µg/mL			99,99 ± 18,18	87,1 ± 10,68 ***	86,14 ± 12,19 ***	107,35 ± 15,59	94,98 ± 14,18

Table S4. Percentages of pre-formed biofilm biomass reduction for each yeast strain in the presence of different concentrations of *Cannabis sativa* resins. A value of 100 corresponds to the maximum reduction of pre-formed biofilm biomass for each strain under the treatment. Asterisks indicate statistically significant differences (*: p < 0,05; **: p < 0,02; ***: p < 0,01).

Yeast	Concentration	Control	DMSO	GEN 2	GEN 3	GEN 5	GEN 6	GEN 7
C. albicans	32 µg/mL	100	100,7 % ± 8,63	57,54 ± 5,44 ***	40,34 ± 6,25 ***	61,9 ± 4,01 ***	25,02 ± 3,84 ***	44,98 ± 8,51 ***
	16 µg/mL			69,34 ± 5,35 ***	62,8 ± 8,29 ***	82,31 ± 15,5 ***	60,21 ± 6,39 ***	51,25 ± 8,67 ***
	8 µg/mL			64,34 ± 6,44 ***	65,53 ± 4,94 ***	85,56 ± 7,33 ***	57,93 ± 12,95 ***	73,2 ± 5,05 ***
	4 µg/mL			78,91 ± 12,19 ***	76,21 ± 5,41 ***	89,78 ± 9,84 ***	66,41 ± 11,54 ***	71,48 ± 10,83 ***
	2 µg/mL			84,84 ± 14,83 ***	70,94 ± 3,96 ***	90,89 ± 5,71 **	70,5 ± 9,39 ***	69,76 ± 10,14 **
	1 µg/mL			93,22 ± 9,36 ***	82,36 ± 12,51 ***	91,68 ± 8,74 **	63,52 ± 4,74 ***	87,67 ± 7,62 **
	0,5 µg/mL			85,88 ± 7,45 ***	86,31 ± 6,76 ***	92,48 ± 6,15 *	67,82 ± 10,58 ***	85,81 ± 8,48 *
	0,25 µg/mL			87,97 ± 14,74 ***	93,13 ± 5,16	93,59 ± 7,5 *	93,85 ± 6,99	90,65 ± 6,38 *
	0,125 µg/mL			99,41 ± 10,29	94,43 ± 5,73	94,06 ± 7,28	100,52 ± 10,51	100,65 ± 10,08
	0,0625 µg/mL			100,84 ± 8,65	93,09 ± 6,69	93,43 ± 6,96	96,22 ± 11,85	104,85 ± 12,07
	0,03125 µg/mL			96,23 ± 8,42	93,97 ± 7,17	97,72 ± 7,61	101,37 ± 12,33	102,33 ± 6,06
S. cerevisiae	16 µg/mL	100	98,42 % ± 2,73	-	-	-	-	58,69 ± 2,87 ***
	8 µg/mL			-	-	-	-	59,28 ± 3,7 ***
	4 µg/mL			36,67 ± 1,83 ***	56,45 ± 2,44 ***	48,05 ± 4,58 ***	39,03 ± 3,2 ***	65,95 ± 17,96 ***
	2 µg/mL			51,87 ± 4,15 ***	56,45 ± 2,44 ***	58,26 ± 4,96 ***	43,75 ± 2,52 ***	60,9 ± 2,86 ***
	1 µg/mL			69 ± 3,79 ***	59,3 ± 4,77 ***	71,16 ± 3,82 ***	64,99 ± 5,9 ***	65,75 ± 18,05 ***
	0,5 µg/mL			80,31 ± 3,35 ***	58,33 ± 2,51 ***	78,65 ± 3,77 ***	70,81 ± 8,94 ***	69,68 ± 12,53 ***
	0,25 µg/mL			80,76 ± 3,98 ***	60,29 ± 2,71 ***	81,51 ± 4,98 ***	76,19 ± 9,8 ***	80,67 ± 18,41 ***

	**0,125 µg/mL**			80,44 ± 3,16 ***	61,41 ± 6,52 ***	86,93 ± 5,64 ***	88,89 ± 6,06 ***	76,16 ± 13,96
	**0,0625 µg/mL**			80,15 ± 3,21 ***	67,6 ± 4,99 ***	87,43 ± 7,81 ***	90,19 ± 9,43 ***	71,3 ± 9,97
	**0,03125 µg/mL**			80,44 ± 3,16 ***	66,57 ± 2,88 ***	89,26 ± 10,28 ***	92,13 ± 15,2 ***	87,19 ± 15,45
	**0,0156 µg/mL**			84,24 ± 3,6 ***	64,93 ± 2,72 ***	87,84 ± 9,97 ***	92,82 ± 7,38 ***	95,13 ± 4,84
	**0,00781 µg/mL**			82,76 ± 3,76 ***	76,18 ± 16,55 ***	89,95 ± 6,75 ***	91,94 ± 12,7 ***	-
	**0,00390 µg/mL**			81,25 ± 3,53 ***	85,59 ± 10,45 ***	92,95 ± 10,14	93,44 ± 3,63 ***	-

Section S2. Statistical and machine learning models

- ***Growth Inhibition***

Note: following the recommendation of Referee 1, the general linear models below were re-fitted with treatment concentration entered as a quantitative covariate on the log2 scale (C = log2 of the two-fold serial dilution series), instead of the raw concentration. Type III sums of squares were used. For reference, the models with untransformed concentration gave R-squared = 84,97 percent (growth inhibition) and 86,59 percent (pre-formed biofilm).
Number of dependent variables: 1
Number of categorical factors: 2
  A=MICROORGANISM
  B=GEN (RESIN)
Number of quantitative factors: 1
  C=log2(CONCENTRATION)
Number of observations: 1980

Analysis of Variance for Absorbance

Source	*Sum of Squares*	*Df*	*Mean Square*	*F-Ratio*	*P-Value*
**Model**	232,3	10	23,23	1800,24	0,0000
**Residual**	25,41	1969	0,01290		

**Total (Corr.)**	257,7	1979			

Type III Sums of Squares

Source	*Sum of Squares*	*Df*	*Mean Square*	*F-Ratio*	*P-Value*
**MICROORGANISM**	149,4	5	29,88	2315,72	0,0000
**GEN (RESIN)**	0,03364	4	0,00841	0,65	0,6256
**log2(CONCENTRATION)**	40,83	1	40,83	3164,12	0,0000
**Residual**	25,41	1969	0,01290		
**Total (corrected)**	257,7	1979			

R-Squared = 90,14 percent
R-Squared (adjusted for d.f.) = 90,09 percent

- ***Pre-formed biofilm***

Number of dependent variables: 1
Number of categorical factors: 2
A=MICROORGANISM
B=GEN (RESIN)
Number of quantitative factors: 1
C=log2(CONCENTRATION)
Number of observations: 1578

Analysis of Variance for Absorbance

Source	*Sum of Squares*	*Df*	*Mean Square*	*F-Ratio*	*P-Value*
**Model**	552,7	10	55,27	1364,01	0,0000
**Residual**	63,50	1567	0,04052		
**Total (Corr.)**	616,3	1577			

Type III Sums of Squares

Source	*Sum of Squares*	*Df*	*Mean Square*	*F-Ratio*	*P-Value*
**MICROORGANISM**	544,9	5	109,0	2689,44	0,0000
**GEN (RESIN)**	4,486	4	1,122	27,68	0,0000
**log2(CONCENTRATION)**	56,81	1	56,81	1401,90	0,0000

**Residual**	63,50	1567	0,04052		
**Total (corrected)**	616,3	1577			

Variance Components

Source	***Estimate***
**Residual**	0,04052

R-Squared = 89,70 percent
R-Squared (adjusted for d.f.) = 89,63 percent

Table S5. Machine learning algorithms and hyperparameters evaluated during the optimization process.

Model family	Explored hyperparameters
Ridge (regularized linear regression)	alpha: 0,01 / 0,1 / 1 / 10 / 100
K nearest neighbors (KNN)	n_neighbors: 3 / 5 / 7 / 11 / 15; weights: uniform / distance
Support vector machine (SVR, RBF kernel)	C: 1 / 10 / 100; gamma: scale / 0,1 / 1; epsilon: 0,01 / 0,1
Decision tree	max_depth: 3 / 5 / 8 / sin límite; min_samples_leaf: 1 / 3 / 5
Random forest	max_depth: no limit/ 12; min_samples_leaf: 1 / 2 / 4; max_features: todas / sqrt
Extra trees	max_depth: no limit/ 12; min_samples_leaf: 1 / 2 / 4; max_features: todas / sqrt

**Model family**	**Explored hyperparameters**
Gradient Boosting	learning_rate: 0,05 / 0,1; max_depth: 2 / 3 / 4
XGBoost	learning_rate: 0,05 / 0,1; max_depth: 3 / 5; subsample: 0,8 / 1,0
LightGBM	learning_rate: 0,05 / 0,1; num_leaves: 15 / 31; min_child_samples: 5 / 20
Neural network (MLP)	capas ocultas: (64,32) / (100); alpha: 0,001 / 0,01

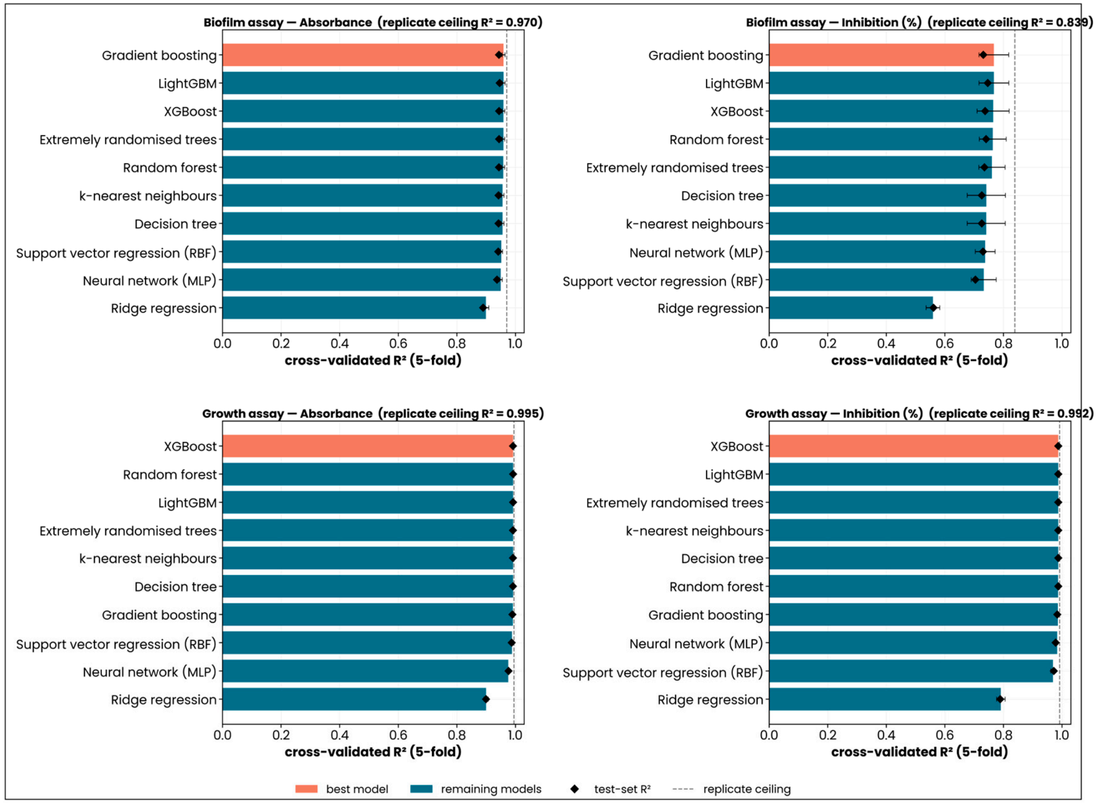

Figure S1. Cross-validation R^2 of the ten model families optimized with GridSearchCV. The orange bar indicates the selected model; the black diamonds, the R^2 in the test set; the dashed line, the ceiling of replicates

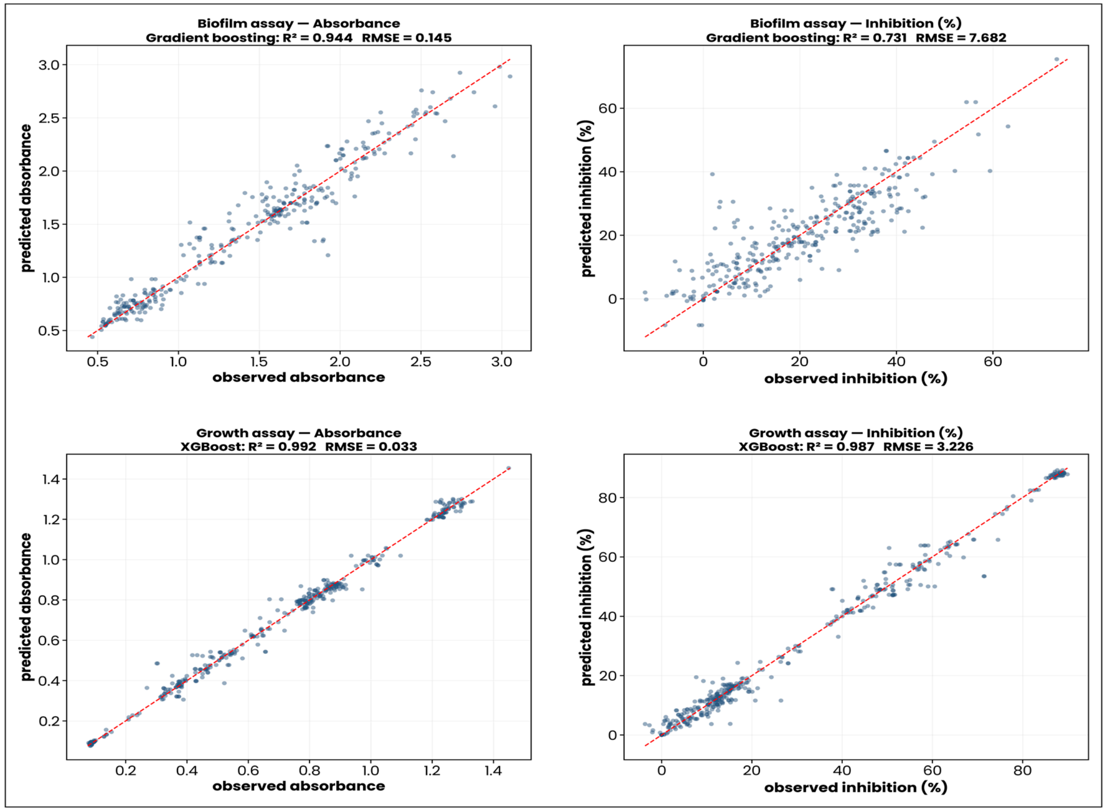

.

Figure S2. Observed vs. predicted values in the test set (20% of the data, not used in training or hyperparameter selection).

Table S6. Results of the Permutation Importance analysis for the selected machine learning models.

Case	Variable	Drop in R^2	DE	Relative Importance
pre-formed biofilm — Absorbance	Microorganismo	2,1822	0,1499	83,8 %
pre-formed biofilm — Absorbance	Gen (resina)	0,0821	0,0100	3,2 %
pre-formed biofilm — Absorbance	Concentración	0,3385	0,0297	13,0 %
pre-formed biofilm — % pre-formed biofilm biomass reduction	Microorganismo	0,6766	0,0591	26,7 %
pre-formed biofilm — % pre-formed biofilm biomass reduction	Gen (resina)	0,3353	0,0401	13,2 %
pre-formed biofilm — % pre-formed biofilm biomass reduction	Concentración	1,5204	0,1132	60,0 %
Growth — Absorbance	Microorganismo	1,4715	0,0848	71,7 %
Growth — Absorbance	Gen (resina)	0,0508	0,0049	2,5 %
Growth — Absorbance	Concentración	0,5299	0,0286	25,8 %
Growth— % inhibition	Microorganismo	1,1587	0,0906	48,7 %

Case	Variable	Drop in R²	DE	Relative Importance
Growth— % inhibition	Gen (resina)	0,1085	0,0105	4,6 %
Crecimiento — % de inhibición	Concentración	1,1142	0,0631	46,8 %

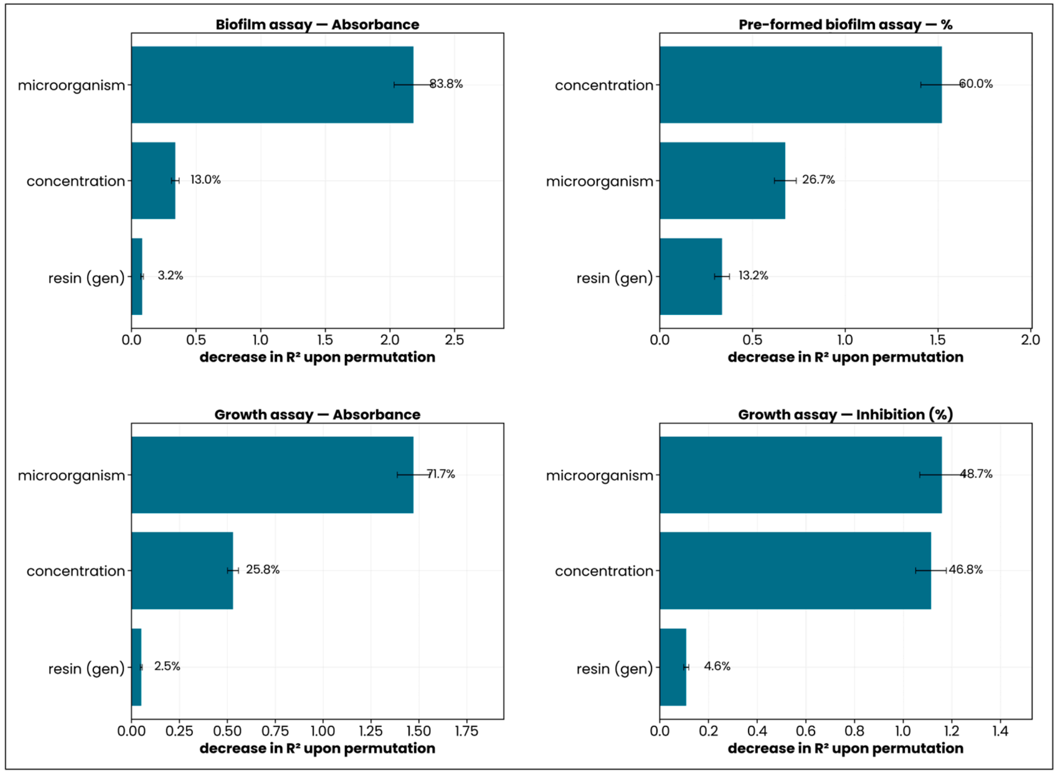

Figure S3. Importance by permutation of the three input variables in each case. The percentage indicates the relative importance within each model.

- ***Importance by category within each variable***

Microorganism

Table S7. Pre-formed biofilm assay -Absorbance

Category	|SHAP| mediun	SHAP effect medium	Average observed value	n
S. aureus	**0,222**	**-0,710**	**0,65**	**246**
P. aeruginosa	0,179	+0,577	2,22	240
E. coli	0,164	-0,508	0,77	246
A. baumannii	0,157	+0,461	2,06	246
S. cerevisiae	0,025	-0,049	1,49	294
C. albicans	0,016	+0,008	1,41	336

Table S8. Pre-formed biofilm assay- % inhibition

Category	|SHAP| mediun	SHAP effect medium	Average observed value	n
S. cerevisiae	2,839	+7,988	23,86	294
S. aureus	2,337	+6,879	18,04	246
A. baumannii	1,074	-3,363	18,57	246
C. albicans	1,035	-2,549	19,42	336
E. coli	0,683	-1,776	20,15	246
P. aeruginosa	0,497	+0,159	23,05	240

Table S9. Growth-Absorbance

Category	|SHAP| mediun	SHAP effect medium	Average observed value	n
C. albicans	0,165	+0,457	1,25	336
S. aureus	0,125	-0,360	0,19	336
S. cerevisiae	0,035	-0,087	0,65	336
A. baumannii	0,024	+0,060	0,77	336
P. aeruginosa	0,006	-0,009	0,65	336
E. coli	0,003	+0,005	0,68	336

Table S10. Growth - % inhibition

Category	|SHAP| mediun	SHAP effect medium	Average observed value	n
S. aureus	13,120	+40,287	74,16	336
S. cerevisiae	2,976	+8,683	26,28	336
C. albicans	1,181	-4,056	13,89	336
A. baumannii	0,849	-0,662	26,94	336
E. coli	0,837	-2,178	25,32	336
P. aeruginosa	0,510	-1,109	26,28	336

Table S11. Pre-formed biofilm assay – Absorbance

Category	|SHAP| mediun	SHAP effect medium	Average observed value	n
GEN 3	0,032	-0,079	1,34	318
GEN 6	0,021	-0,034	1,40	312
GEN 5	0,019	+0,044	1,50	318
GEN 7	0,012	-0,015	1,43	324
GEN 2	0,009	+0,013	1,46	306
CONTROL	0,001	+0,012	1,78	30

Table S12. Pre-formed biofilm assay - % inhibition

Category	|SHAP| mediun	SHAP effect medium	Average observed value	n
GEN 3	1,496	+3,819	25,77	318
GEN 5	1,065	-2,500	17,62	318
GEN 6	0,935	+0,625	20,95	312
GEN 2	0,770	-1,973	17,31	306
GEN 7	0,699	+1,359	22,85	324
CONTROL	0,013	-0,257	0,00	30

Table S13. Growth — Absorbance

Category	|SHAP| mediun	SHAP effect medium	Average observed value	n
GEN 2	0,009	-0,009	0,69	396
GEN 6	0,009	-0,002	0,69	396
GEN 3	0,003	+0,002	0,70	396
GEN 7	0,003	0,000	0,70	396
CONTROL	0,002	+0,054	0,99	36
GEN 5	0,002	-0,001	0,70	396

Table S14. Growth — % Inhibition

Category	|SHAP| mediun	SHAP effect medium	Average observed value	n
GEN 2	0,995	+0,871	33,25	396
GEN 6	0,943	+0,234	32,70	396
GEN 7	0,327	+0,188	32,66	396
GEN 3	0,309	+0,012	32,38	396
CONTROL	0,217	-4,626	0,00	36
GEN 5	0,217	+0,229	32,66	396

Concentration

Table S15. Pre-formed biofilm % assay

Concentration level	|SHAP| medium	SHAP effect medium	% inhibition observed	n
**32**	**33,47**	**+33,47**	**50,82**	**36**
0.00195312	19,85	-19,85	6,08	30
0 (control)	18,62	-18,62	0,00	30
0.00390625	16,00	-16,00	10,98	60
16	15,81	+15,81	34,22	108

Concentration level	|SHAP| mediun	SHAP effect medium	% inhibition observed	n
8	13,46	+13,46	31,51	120
0.0078125	11,62	-11,62	15,06	60
0.03125	11,20	-11,20	12,10	90
0.015625	9,73	-9,73	18,61	60
4	9,57	+9,57	26,95	120
0.0625	9,30	-9,30	14,81	90
0.125	8,69	-8,69	10,19	180
2	6,97	+6,97	27,18	144
1	3,60	+3,60	22,96	150
0.25	2,77	-1,98	18,35	180
0.5	0,82	-0,08	18,13	150

Table S16. Growth - % inhibition

Concentration level	|SHAP| mediun	SHAP effect medium	% inhibition observed	n
**256**	**34,02**	**+34,02**	**69,83**	**120**
128	27,56	+27,56	62,86	120
0 (control)	26,42	-26,42	0,00	36
64	24,70	+24,70	59,90	120
0.25	22,42	-22,42	9,24	180
0.03125	19,94	-19,94	9,46	60
0.0625	19,19	-19,19	10,12	60
0.125	18,61	-18,61	10,81	60
32	17,80	+17,80	48,22	180
0.5	13,68	-13,68	17,80	180

Concentration level	|SHAP| mediun	SHAP effect medium	% inhibition observed	n
1	10,96	-10,96	20,56	180
16	7,83	+7,83	39,02	180
2	7,57	-7,57	24,14	180
4	4,19	-1,76	30,14	180
8	3,93	+0,35	32,39	180

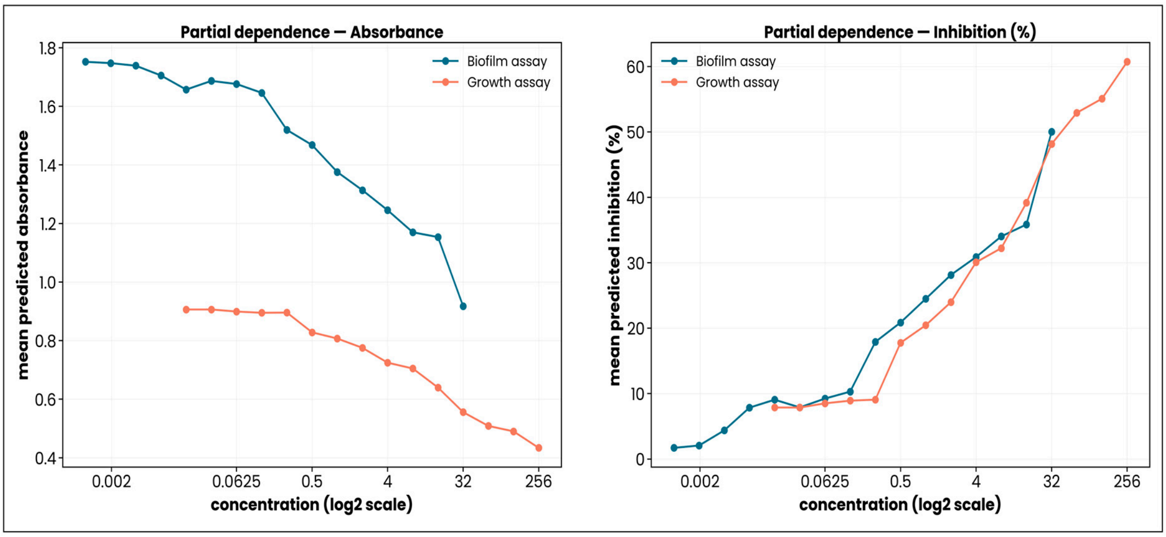

Figure S4. Partial concentration dependence: mean predicted response by fixing the concentration at each level and averaging over all other combinations.

Table S17. Botanical identification, regulatory registration, geographical origin, and cultivation characteristics of the *Cannabis sativa* L. cultivars used to produce the evaluated resins.

Resin code	Cultivar	ICA registration	Geographical origin	CBD in source flower (%)	THC in source flower (%)	Propagation	Cultivation condition	Planting density	Photoperiod (vegetative / flowering)	Transplant-to-harvest (days)
GEN2	ANDICA CBD	CANN-21-0429	Manizales, Colombia	11.79	0.42	Asexual	Greenhouse	1 plant/m²	18 h/6 h / 12 h/12 h	109
GEN3	EMBERA	CANN-21-0430	Mistrató, Risaralda, Colombia	12.05	0.41	Asexual	Greenhouse	1 plant/m²	18 h/6 h / 12 h/12 h	110
GEN5	YOCOTO	CANN-21-0433	Yotoco, Valle del Cauca, Colombia	13.10	0.48	Asexual	Greenhouse	1 plant/m²	18 h/6 h / 12 h/12 h	109
GEN6	LIMON CBD	CANN-21-0431	Belén de Umbría, Risaralda, Colombia	17.97	0.67	Asexual	Greenhouse	1 plant/m²	18 h/6 h / 12 h/12 h	110
GEN7	VALLE DE LOS UMBRAS	CANN-21-0432	Belén de Umbría, Risaralda, Colombia	12.43	0.43	Asexual	Greenhouse	1 plant/m²	18 h/6 h / 12 h/12 h	110

Notes. CBD and THC values correspond to the characterization of the source flowers reported in the available official cultivar documentation and were determined by GC-FID; they should not be interpreted as the cannabinoid composition of the processed resins analyzed by HPLC-PDA in the present study. The available documentation classifies all source cultivars as non-psychoactive, CBD-predominant cannabis. No herbarium voucher specimen or voucher number for the source plant material was identified in the official documentation available to the authors. Cultivar type (variety or hybrid), genealogy, breeding methodology, soil type, specific geographical coordinates, and exact collection date/season were not available in the reviewed documentation.

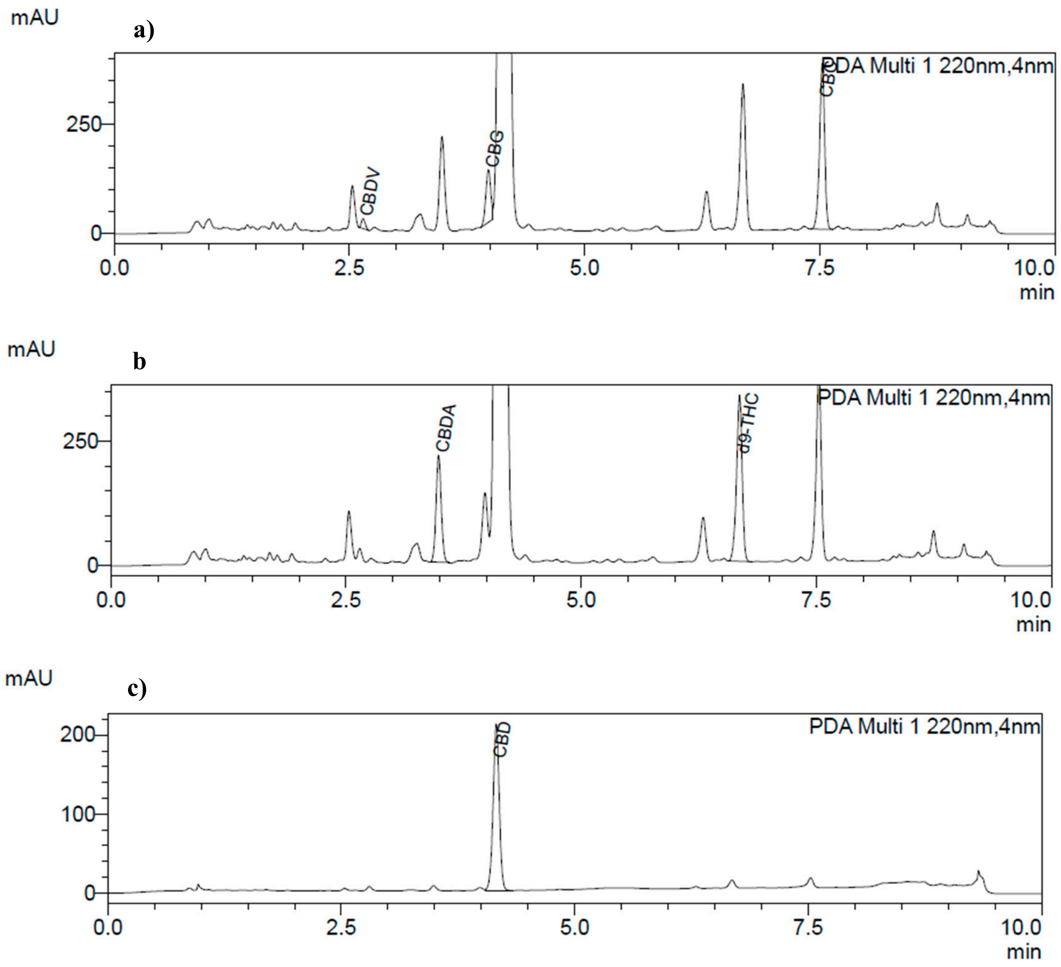

Figure S5. HPLC-PDA chromatogram of cannabinoid profile **a)** CBDV, CBG, and CBC, **b)** CBDA and Δ^9-THC and c) cannabidiol (CBD) in the diluted resin sample for GEN2.

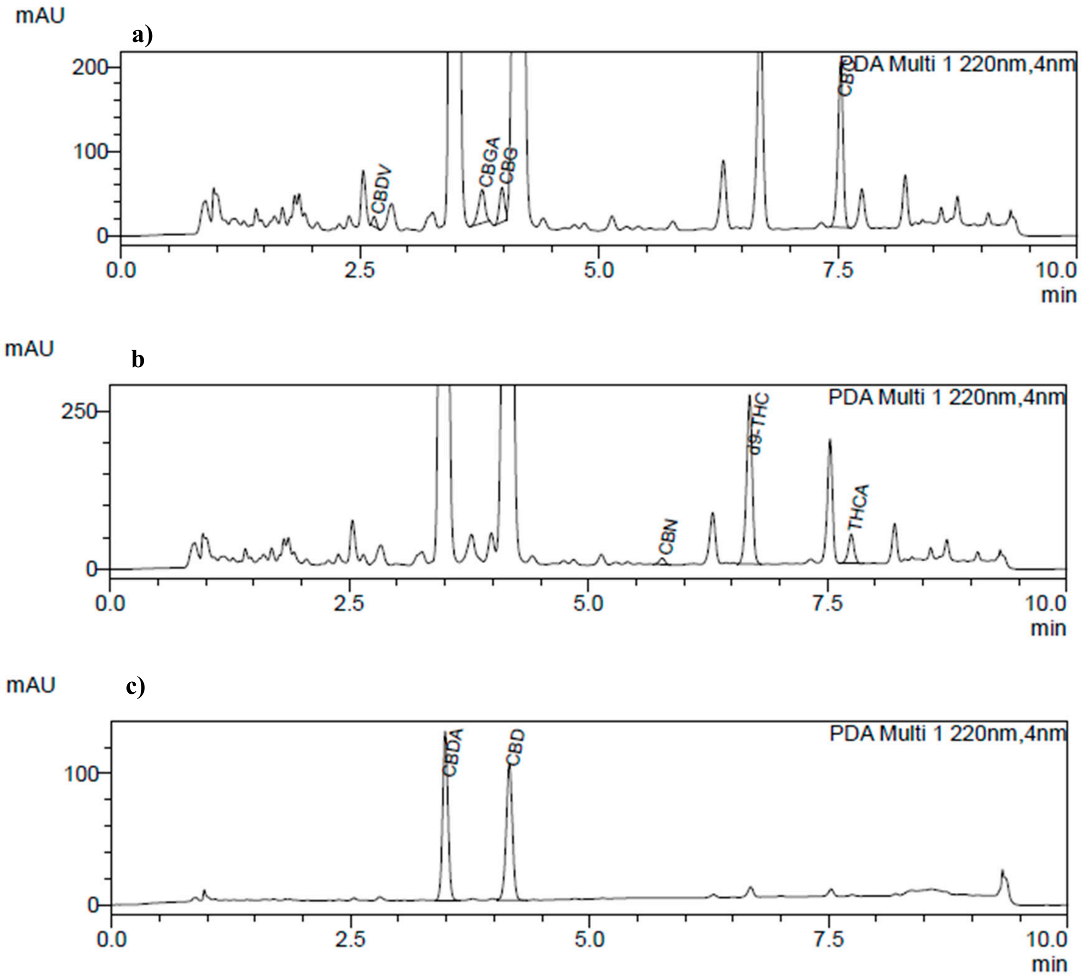

Figure S6. HPLC-PDA chromatogram of cannabinoid profile **a)** CBDV, CBG, and CBC, **b)** CBDA and Δ^9-THC and c) cannabidiol (CBD) in the diluted resin sample for GEN3.

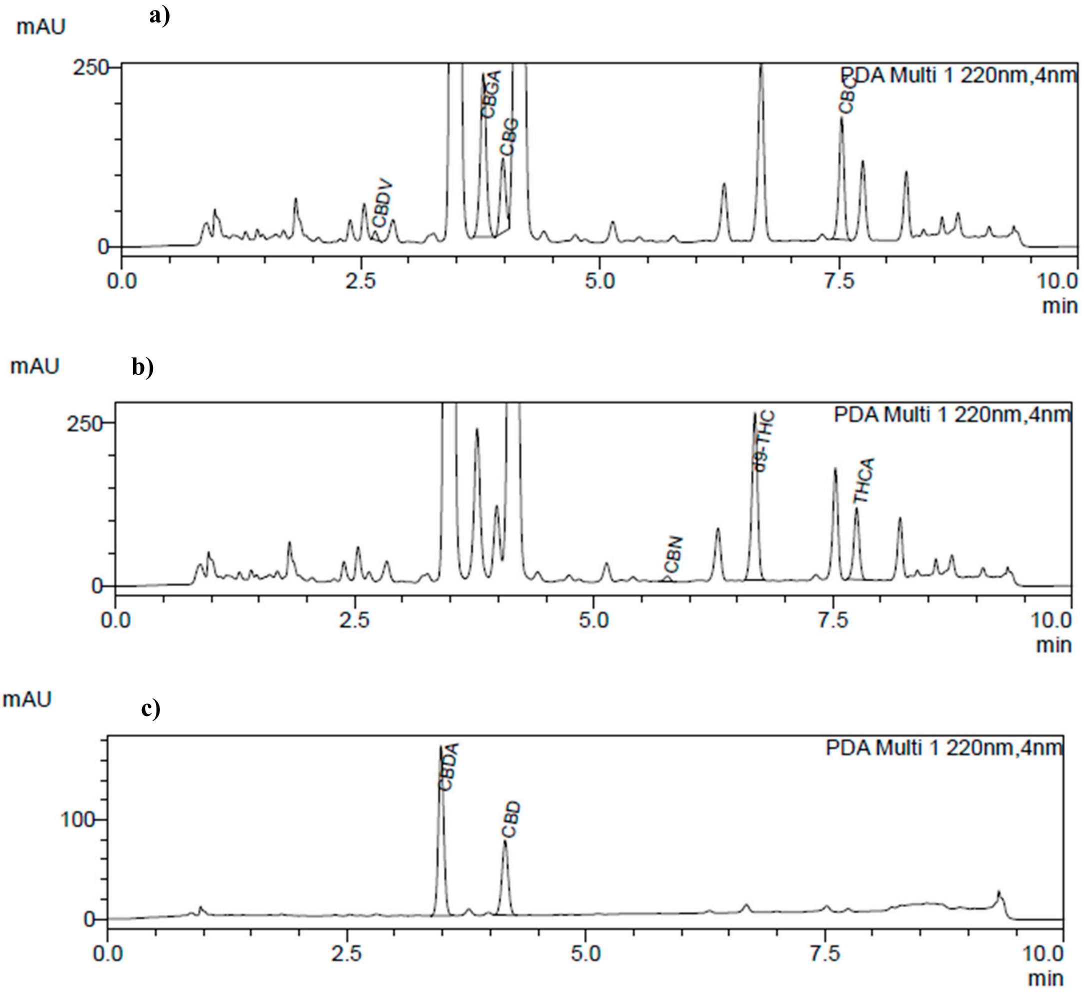

Figure S7. HPLC-PDA chromatogram of cannabinoid profile **a)** CBDV, CBG, and CBC, **b)** CBDA and Δ^9-THC and c) cannabidiol (CBD) in the diluted resin sample for GEN5.

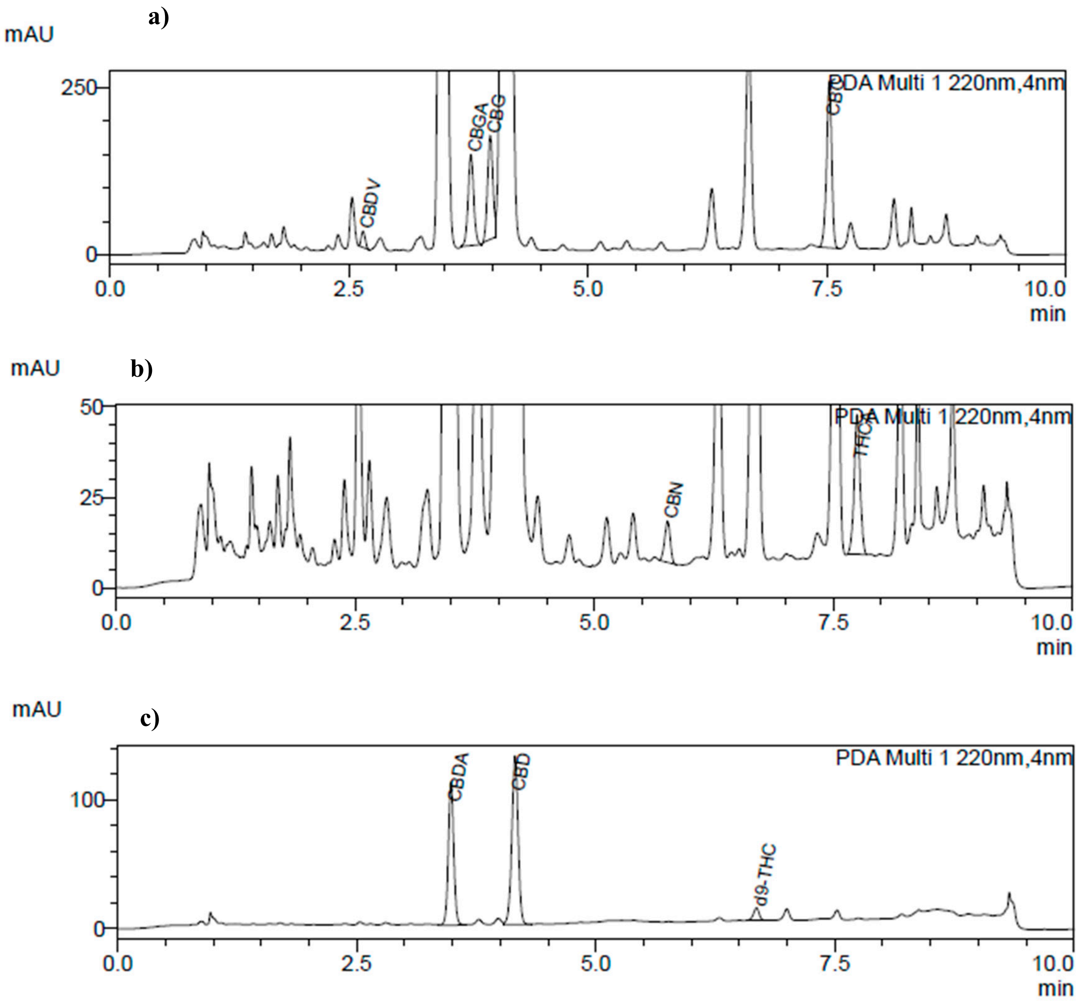

Figure S8. HPLC-PDA chromatogram of cannabinoid profile **a)** CBDV, CBG, and CBC, **b)** CBDA and Δ^9-THC and c) cannabidiol (CBD) in the diluted resin sample for GEN6.

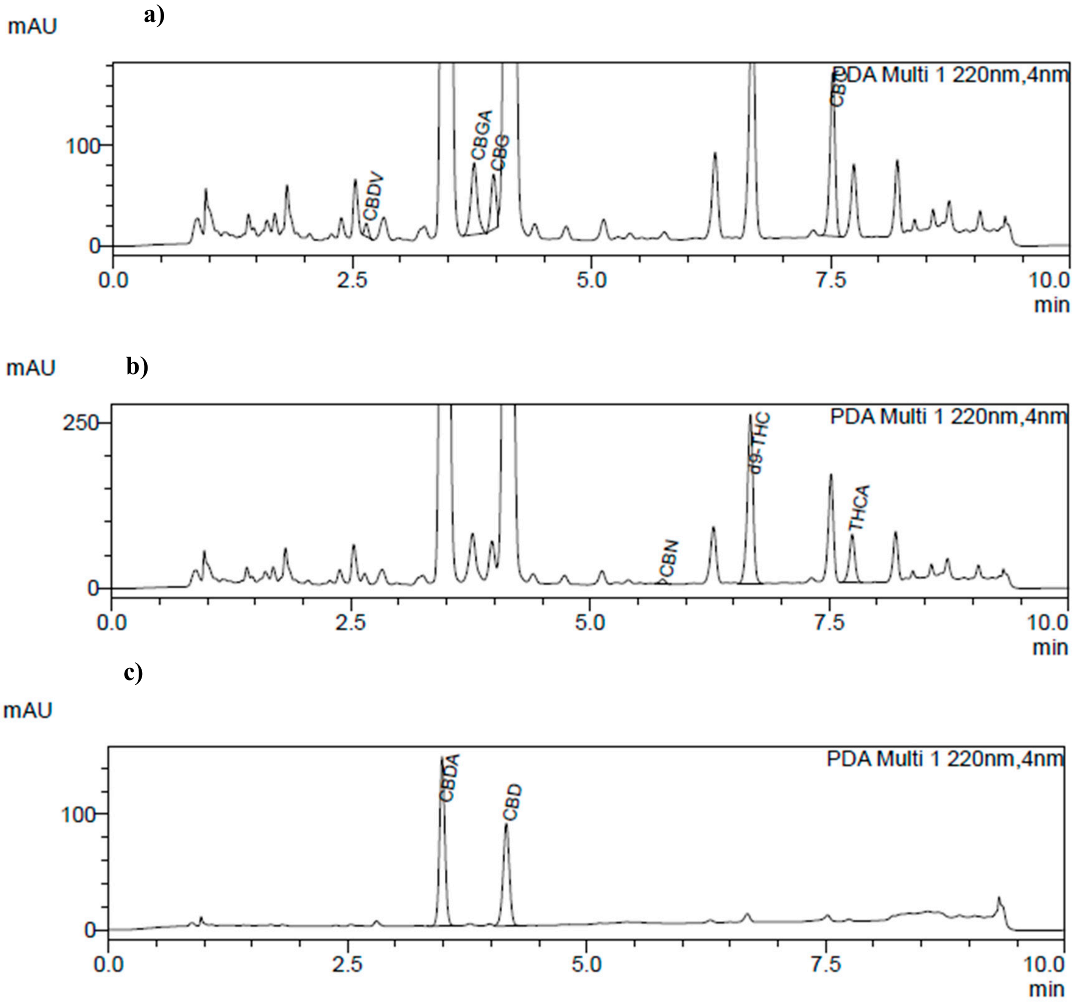

Figure S9. HPLC-PDA chromatogram of cannabinoid profile **a)** CBDV, CBG, and CBC, **b)** CBDA and Δ^9-THC and c) cannabidiol (CBD) in the diluted resin sample for GEN7.
